# Key points for the development of antioxidant cocktails to prevent cellular stress and damage caused by reactive oxygen species (ROS) during manned space missions

**DOI:** 10.1038/s41526-021-00162-8

**Published:** 2021-09-23

**Authors:** Xavier Gómez, Serena Sanon, Kevin Zambrano, Samira Asquel, Mariuxi Bassantes, Julián E. Morales, Gabriela Otáñez, Core Pomaquero, Sarah Villarroel, Alejandro Zurita, Carlos Calvache, Kathlyn Celi, Terry Contreras, Dylan Corrales, María Belén Naciph, José Peña, Andrés Caicedo

**Affiliations:** 1 Universidad San Francisco de Quito USFQ, Colegio de Ciencias de la Salud, Escuela de Medicina, Quito, Ecuador; 2 Universidad San Francisco de Quito USFQ, Instituto de Investigaciones en Biomedicina iBioMed, Quito, Ecuador; 3 Cornell University, Ithaca, NY USA; 4 Mito-Act Research Consortium, Quito, Ecuador; 5 School for Mental Health and Neuroscience (MHeNs), Maastricht University, Maastricht, The Netherlands; 6 Sistemas Médicos SIME, Universidad San Francisco de Quito USFQ, Quito, Ecuador

**Keywords:** Risk factors, Proteolysis, Translational research, Diagnostic markers

## Abstract

Exposure to microgravity and ionizing radiation during spaceflight missions causes excessive reactive oxygen species (ROS) production that contributes to cellular stress and damage in astronauts. Average spaceflight mission time is expected to lengthen as humanity aims to visit other planets. However, longer missions or spaceflights will undoubtedly lead to an increment in microgravity, ionizing radiation and ROS production. Strategies to minimize ROS damage are necessary to maintain the health of astronauts, future space colonists, and tourists during and after spaceflight missions. An antioxidant cocktail formulated to prevent or mitigate ROS damage during space exploration could help maintain the health of space explorers. We propose key points to consider when developing an antioxidant cocktail. We discuss how ROS damages our body and organs, the genetic predisposition of astronauts to its damage, characteristics and evidence of the effectiveness of antioxidants to combat excess ROS, differences in drug metabolism when on Earth and in space that could modify antioxidant effects, and the characteristics and efficacy of common antioxidants. Based on this information we propose a workflow for assessing astronaut resistance to ROS damage, infight monitoring of ROS production, and an antioxidant cocktail. Developing an antioxidant cocktail represents a big challenge to translate current medical practices from an Earth setting to space. The key points presented in this review could promote the development of different antioxidant formulations to maintain space explorers’ health in the future.

## Introduction

“We choose to go to the Moon in this decade and do other things, not because they are easy, but because they are hard…,” John Fitzgerald Kennedy, September 12, 1962^1^.

After 60 years of space exploration, JFK’s speech still resonates today, motivating all fields of science to face the many challenges to colonize space. With an extended presence outside Earth’s atmosphere, astronauts are exposed to many detrimental factors to their health that are starting to be understood and must be resolved in order to maintain health during longer missions. The history of space exploration is characterized by a series of technological advancements leading to better life support systems, each one of them allowing longer missions outside Earth. However, strategies or preventive measures to maintain health and avoid pathologies caused by extended journeys into space still need to be explored and developed.

In 1961, Yuri Gagarin became the first human to go into outer space, with an orbital flight duration of about 100 min^2^. Seven years later, Neil Armstrong became the first man to set foot on the moon with a mission time of 8 days^3,4^. Since the first spaceflight mission in 1961, >500 humans have been sent to space, some on shorter trips that lasted <1 month, others on longer journeys that were up to 1 year long^5^. In the near future, the National Aeronautics and Space Administration (NASA) plans to launch the Artemis program, which will send the first female astronauts to the moon^6^. With it, NASA plans to look further into human spaceflights, such as manned missions to Mars. This means an increase in spaceflight time and complexity, as it takes 7 months to get to the red planet^6,7^.

Outer space is hazardous to life. When our bodies are driven out of Earth’s comfort zone, homeostatic processes are disrupted, and these disruptions are exacerbated with time. In 2019, scientists from NASA published a study that analyzed various physiological markers in a pair of twins, one of whom spent a year in space while the other remained on Earth^5^. The study indicated that astronauts could experience mitochondrial dysfunction, immunological stress, and alterations in the telomere length, among other physiological adaptations, from spending a year in space^5^. There are also concerns regarding the effects of space after an astronaut returns to Earth conditions, in regard to damage accumulated in the cardiovascular, ocular, and cognitive systems, in addition to molecular pathways within cells^5^. In 2015, NASA published a health hazard report of astronauts in space and identified five main hazards of spaceflight: isolation, hostile environment, limited resources, radiation, and altered gravity (microgravity)^8^. The latter two generate cell metabolic stress and consequently lead to an increase in reactive oxygen species (ROS)^9^. ROS accumulation causes oxidative stress that can damage lipids, proteins, and ribonucleic acids^10^. Furthermore, ROS induces cell death^11^, cell senescence, repair failure^12^, inhibition of cell proliferation, and cell cycle arrest^13^. Fortunately, the large quantities of free radicals produced by ROS can be attenuated by antioxidants.

Antioxidants are compounds that have a role in the inhibition of oxidative molecules that degrade cellular structures that could cause disease^14^. It is important to point out that a causal association between a reduced risk of illness and antioxidant consumption is still missing and more placebo-controlled interventions are necessary to justify antioxidant supplementation^15–19^. However, environmental conditions on Earth are very different from those found in space and the human body may need extra antioxidants in order to prevent ROS damage besides those provided by the spacecraft menu. These antioxidants could be supplemented in the food provided to astronauts or as a capsule cocktail.

Developing a balanced diet for astronauts that is rich in antioxidants and other nutrients is a priority for any space agency. Designing a menu to maintain balanced vitamin content in packaged food taken to orbit is a challenge as the freeze drying process can cause food to lose some of its nutritional value and taste^20^. Food with radioprotective properties after digestion could be necessary to mitigate the damaging effects of space radiation (radioprotective nutrition)^20,21^. Resupplying fresh fruits and vegetables to astronauts becomes difficult during long-term and distance missions^20,21^. Until more diverse food options that have a high nutritional content and a good taste are developed, the supplementation of vitamins such as A, C, and E is necessary to maintain optimal levels.

There are two types of antioxidants: endogenous and exogenous. Our body produces natural antioxidants such as superoxide dismutase (SOD), catalase (CAT), glutathione peroxidase (GPx), and glutathione (GSH)^14^. When ROS levels increase excessively, exogenous antioxidants might be needed such as vitamin A, vitamin C, vitamin E, carotenoids, and polyphenols^14^. ROS can have negative effects on various processes, including redox regulation, cell signaling, promotion of proliferation, immunity, apoptosis, autophagy, and necrosis^22^. However, ROS, while being detrimental in excess, does have a physiological role in cell differentiation, immune cell activation, metabolic adaptation, and autophagy. Thus, it has been proposed that their presence is not detrimental and could promote the maintenance of health^15,23^. Therefore, while an ideal antioxidant mix should reduce ROS, it is important to ensure that an optimal amount of ROS remains^14^. Thus, moderation is crucial as we consider antioxidants as preventive or therapeutic agents for ROS damage.

Astronauts are exposed to 100 times more ionizing radiation than the general population, which causes an abnormally high level of ROS production in their cells^24^. Ionizing radiation from galactic cosmic rays, solar particle events, and trapped belt radiation represents a major health concern that has not been completely understood or estimated in deep spaceflight missions^25,26^. There have been proposed methods for enhancing human radioresistance, such as upregulating endogenous repair and radioprotective mechanisms by gene therapy, and substituting organic molecules with stronger isoforms that may resist deep space radiation^26^. We could speculate that, with the latest advancement of RNA therapeutics in the form of vaccines that involve mRNAs coding for different proteins or long peptides^27–29^, it would be possible to induce the transient production of proteins with radioprotective properties and mitigate the damage caused by radiation during deep space missions. However, until methods are developed for radioresistance, creating an antioxidant cocktail for human spaceflight could be paramount to prevent and reduce the cellular damage suffered by astronauts. In this review, we provide key aspects to consider regarding the development of an antioxidant cocktail that could be used to prevent cellular stress and damage during spaceflight. First, we explain what ROS are and why it is important to develop an antioxidant cocktail. Later, we introduce the significance of identifying astronauts with a higher genetic susceptibility to ROS damage by using genome-wide association studies (GWAS). This would allow for the identification of polymorphisms carried by space explorers that may help them maintain good health or affect their specific antioxidant cocktail supplementation. Then we detail how ROS affects various organ systems within astronauts, ranging from the musculoskeletal to the immune system. Important aspects regarding the differences between antioxidant administration on Earth and in space and their effectiveness in various diseases will also be discussed. Based on this information, a workflow is proposed for assessing astronaut resistance to ROS damage, infight monitoring of ROS production, and an antioxidant cocktail. It is our goal to provide key aspects to consider when developing antioxidant cocktails that will be of great value when traveling to space.

### ROS and the importance of developing antioxidant cocktails

The human body is not well adapted to high concentrations of ROS, as this can cause damage to proteins, lipids, carbohydrates, and DNA across all organ systems^30^. ROS are radical and non-radical oxygen species produced by the partial reduction of oxygen and include species such as hydrogen peroxide (H_2_O_2_), hydroxyl radical (HO), and the superoxide anion (O_2_^−^)^11^. ROS are produced in the electron transport chain, NADPH oxidase-dependent, and xanthine oxidase-dependent pathways^31^. The superoxide anion is mainly produced by complexes I and III in mitochondria^31^. For instance, an excess of ROS has the ability to damage lipids, proteins, and DNA, resulting in mitochondrial stress, followed by apoptosis and cell death^32^. Both astronauts and those on Earth can suffer from cell and tissue damages induced by ROS^33^.

Antioxidants act as reducing agents, meaning they have the ability to donate some of their electrons to other molecules, particularly to free radicals created after an oxidation process^32^. Chemical reactions between antioxidants and free radicals constantly occur within the human body and are essential for the maintenance of homeostasis, as ROS are unfavorable to the human biological system^22^. Antioxidants prevent these harmful consequences by stabilizing free radicals and avoiding oxidative stress^32^. There are several enzymes that counteract damage by ROS, including GPx, SOD, GSH, and CAT, by decreasing the concentrations of the most harmful oxidants in the tissues^34^.

**Fig. 1:**
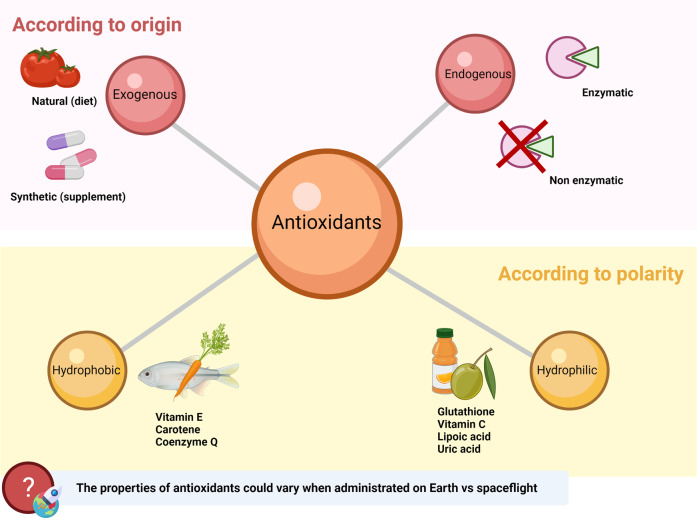
Antioxidant categorization: enzymatic endogenous, non-enzymatic endogenous, and exogenous. Created with BioRender.com.

Antioxidants can be categorized into three groups: enzymatic endogenous, non-enzymatic endogenous, and exogenous^35^ (Fig. 1). Endogenous antioxidants are by-products of metabolism within the body, while exogenous antioxidants are consumed through the diet^36^. Exogenous antioxidants can either be consumed through the intake of fruits and vegetables (natural antioxidants) or of chemically produced synthetic antioxidant supplements. A synergistic relationship between endogenous and exogenous antioxidants works to return the body to redox homeostasis^36^. Additionally, antioxidants can be further classified as hydrophilic or hydrophobic^22^. Common hydrophilic antioxidants include GSH, vitamin C, lipoic acid, and uric acid, while hydrophobic antioxidants include vitamin E, carotenes, and coenzyme Q^22^. Despite their beneficial effects, not all of these compounds have the same effects and are able to be administered orally. Their protection against free radicals varies according to their ability to form redox cycles, which depends on the individual’s metabolism and genetics as well as the amount administered^22^. Furthermore, the properties of each antioxidant could vary when administered on Earth versus during spaceflight.

Despite their beneficial effects, research has indicated that exogenous antioxidants in high doses, and in the presence of metal ions, can cause pro-oxidant effects^35^. Studies have also suggested that high doses of some antioxidants, such as quercetin and flavonoids, reduce cell survival^35,37,38^. Thus, the concentration of these substances is crucial to determine whether they will have oxidative or antioxidative properties. In addition, the different optimal concentration of each antioxidant must be taken into consideration before administration. However, more studies are needed to find out whether the concentrations of antioxidants used on Earth should be the same in space. For example, the maximum daily recommended dose for vitamin C and vitamin E is 2000 mg and 1000–1200 mg, respectively^39–41^, but these concentrations may need to be modified for consumption during spaceflight.

As mentioned before, effects of antioxidants are beneficial for the body because they counteract the detrimental effects of free radicals produced during cellular metabolism. However, over-administration of these compounds can also give rise to adverse side effects^39^. While large amounts of ROS are harmful, the body does need a baseline level of ROS to aid in important cellular functions, such as cell signaling and redox regulation^14^. Thus, an excess consumption of antioxidants such as vitamin C and E (dose mentioned above) could cause cell damage and harm many cellular processes based on clinical trial information^14,40^. The most common consequences for vitamin C excess include diarrhea, nausea and vomiting, stomach hyperacidity, intestinal colic, and insomnia^39^. Excess vitamin E is very rare, but it can cause diarrhea, nausea, fatigue, and even increase the risk of strokes^39^. In addition, an excess of carotenes (vitamin A) can lead to nausea, vomiting, headaches, dry skin, erythematous-desquamative pathologies, hair loss, and even liver diseases^39^. The human antioxidant defense is complex as it must minimize the levels of ROS while still allowing a baseline amount of ROS to perform cell signaling and redox regulation^14^.

The health of astronauts is evaluated before, during, and after spaceflights. However, direct evaluation of endogenous and exogenous antioxidant levels, to our knowledge, are not routinely included in these health tests. Space Flight Participants (SFP), also known as Space Tourists, must be evaluated for their eligibility for short-duration spaceflight to the International Space Station (ISS). The criteria applied to SFP is less rigorous than those for professional astronauts designated for long-term missions at the ISS, as SFPs are typically fare-paying tourists who do not have operational responsibilities^42^. Since 1959, the health monitoring of astronauts and cosmonauts has been rigorous in order to rule out latent diseases and evaluate physiological, functional, and reserve potential regarding future spaceflight performance. Tests include physical examination, laboratory analysis (complete hematology workup, urinalysis, serologic test, glucose tolerance test, acid and alkaline phosphatase, blood urea nitrogen, sodium, serum glutamic-oxaloacetic transaminase, glutamic pyruvic transaminase, total protein with albumin and globulin, separate determination of Alpha 1, Alpha 2, and globulins, among others), urine and fecal samples, ultrasound evaluations and X-rays of most of the body parts^43^. During inflight examination, astronauts are subjected to many tests, including orthostatic intolerance, physical deconditioning, psychological stress, trauma, functional exercise capacity, blood pressure by the tachoscillographic method, kinetocardiogram, sphygmogram of the carotid, radial, and femoral arteries, and viral or bacterial infections that may have been acquired before flight but are manifested during the mission. Health is estimated on daily reports and weekly cumulative biomedical data. Complete medical examinations are performed 2 weeks before returning to Earth. These tests are designed to predict astronaut health, risk of disease, and monitoring after postflight readaptation^43^. Based on the available information, astronauts’ health tests are part of the Medical Examination Requirements (MER) and annual Lifetime Surveillance of Astronaut Health (LSAH) examination^44–46^. These tests are used to estimate the overall health of astronauts and to take preventive measures if needed. MER and LSAH tests evaluate any possible negative effects that may result from the abnormal accumulation of ROS; however, ROS or antioxidants are not directly measured. MER considers hematology, blood biochemistry, urinalysis, musculoskeletal, dermatology, ophthalmology, audiology, cardiopulmonary, gastroenterology, reproductive, and behavioral health^44^. LSAH screens and monitors occupational related injury or disease. Even if symptoms of physiological damage could be identified by continuous medical examinations like MER and LSAH, the predisposition of each astronaut to space environmental harm should be evaluated in order for preventive measures to be taken, such as antioxidant supplementation. The risk for chronic diseases in astronauts is constantly evaluated and the parameters of evaluation routinely updated to modify medical standards that allow better care^43,47^.

There are few research reports regarding testing astronaut levels of antioxidants before missions. It is important to note, however, that astronauts from the ISS expeditions 1–8 were tested for oxidative stress upon landing, and researchers found that urine concentration of 8-hydroxy-2′-deoxyguanosine (8OHdG) was elevated by 32% upon landing on Earth, despite no evidence of lipid peroxidation, which is an indication of an increase in damaged DNA^48^. 8OHdG is one of the primary oxidative modifications in DNA caused by hydroxylation of deoxyguanosine residues, which are then excised and travel along the circulatory system to finally be secreted in urine^49^. Thus, blood 8OHdG levels are markers of oxidative DNA damage^49^. Additionally, astronauts were also tested for red blood count SOD that was found to be reduced, thus suggesting a decreased antioxidant capacity^48^. They were also tested for GSH reductase, GPx, β-Carotene, folate, vitamin D and E, retinol, among others^48^. Vitamin D supplement was given during the flight but serum 25- hydroxycholecalciferol was decreased after flight^48^. The authors suggested that the metabolism of vitamin D or its function could have been altered^48^. In another study, researchers also found a reduction of blood antioxidants as well as an increased amount of lipid peroxidation in humans after long-term spaceflight in Russian cosmonauts and rats^50–52^. During and after spaceflight, there is an increase of 8-iso-prostaglandin F2α and 8OHdG, which can be used for markers of damage to lipids and DNA^50^. More research on the endogenous and exogenous antioxidant levels of astronauts could be useful in the procurement of data and lead to the improvement of preventive interventions.

Natural human antioxidant defenses are not always sufficient to maintain a proper ROS balance. Therefore, it is important to consider the development of an exogenous antioxidant cocktail that can supplement the antioxidants produced by the body to diminish the negative effects of ROS. As astronauts accumulate excess ROS in space due to microgravity and radiation, the administration of antioxidants may help increase astronaut health by mitigating this process. The different properties of each antioxidant should be taken into account in order to create an optimal cocktail that can attenuate the reduced length and quality of life astronauts face due to their time in space, while keeping ROS concentrations above the minimally viable level. An antioxidant load could be administered in periodic schedules before and during an astronaut’s time in space to achieve a redox balance that maintains homeostasis and reduces the harmful effects of cellular stress in space.

The development of countermeasures, such as an antioxidant cocktail to prevent the stress and damage produced by ROS in astronauts, is key to counteracting the development of cancer and chronic diseases for future space travelers^47^. Numerous combinations of antioxidants have already been tested in vitro and in vivo. For example, antioxidants such as *N*-acetyl cysteine, ascorbic acid (or vitamin C), coenzyme Q10, folic acid, GSH, α-lipoic acid, niacin, L-selenomethionine (L-SeMet), thiamin, and vitamin E succinate have been tested and show promise^47^. These antioxidants protect cellular structures such as the cell membrane and DNA in addition to improving endogenous anti-ROS defense mechanisms^47^. The understanding of the genetic predisposition of individuals to ROS damage is key for the personalized administration and adequate use of antioxidants.

Even if candidate antioxidant cocktails have been proposed based on the scientific knowledge of their effects, their translation to the field, and specifically to astronauts, has not been successful^47,53,54^. In our opinion, the lack of success in the development of preventive antioxidant treatments for astronauts may be due to the absence of prior genetic testing that determines each astronaut’s capacity to produce endogenous antioxidants. This process needs to be carefully considered when determining proper exogenous antioxidant administration. Additionally, the understanding of how ROS affects our physiology should be extended to multiple systems, as targeted antioxidant therapy for specific tissues is currently limited. The difference in pharmacodynamics and pharmacokinetics of antioxidants and other compounds when administered on Earth or in space could be misleading. This information would help in the transition of antioxidant cocktail administration from in vitro to in vivo and then to clinical applications.

### GWAS to assess the susceptibility of astronauts to ROS damage

An excess of ROS in our cells and organs has been related to the development of a variety of pathologies^55^. However, in healthy physiological states, ROS also serve as second messengers, modulating the transduction of many signaling pathways, especially the innate immune response^56^. Our cells have the capacity to balance excess ROS formation and detoxification with the coordinated expression of genes encoding antioxidant proteins to prevent protein oxidation and DNA damage^56^. Assessing the expression of self-produced and regulated antioxidant proteins in astronauts could help identify space travelers who may need different types and concentrations of antioxidant supplementation.

It has been observed that an excess of ROS activates the nuclear factor-kappa B (NF-κB), NF-E2-related factors (NRF1, 2), and small musculoaponeurotic fibrosarcoma (sMAF) proteins in human cells. These regulatory factors stimulate important genes related to ROS regulation, such as SOD2 and ferritin heavy chain (FTH1)^56–59^. The NRF2/sMAF protein complex regulates the *cis*-acting antioxidant response elements (ARE)^60^ in genes such as GSH *S*-transferase, c-glutamyl cysteine synthetase, NAD(P)H quinone reductase (NQO1), heme-oxygenase-1 (HO-1), and thioredoxin 2 (Trx2)^56^. ARE have additional regulatory elements that provide selectivity toward diverse transcription factors^60^. Understanding how polymorphisms in ARE prevent the development of ROS-related diseases is key for determining the effective dosage of antioxidants and time of administration. This could also lead to stimulation of ARE to help prevent biological aging both on Earth and in space.

Identifying genetic variants that may play a role in the predisposition of higher ROS damage and biological aging both on Earth and in space is key to administering antioxidant-preventive strategies. GWAS have identified >2000 single-nucleotide polymorphisms (SNPs) associated with human diseases^61^. SNPs in NRF2/sMAF-binding sites are rare in humans as they are susceptible to negative selection. However, SNPs that enhance binding of transcription factors such as NRF2/sMAF promote a positive regulation of antioxidant proteins that are of great interest for the understanding of human predisposition to biological aging^61^. It has been shown that eight polymorphic ARE are linked to a higher disease risk in individuals of European ancestry. One of these SNPs (rs242561) was located in the regulatory region of the microtubule-associated protein Tau gene, which induced a strong binding of the NRF2/sMAF complex, leading to increased transcription and a reduced risk of parkinsonian diseases^61,62^. Even if the overexpression and activity of NRF2 could be involved in positive antioxidant effects, it is of great importance to understand the effects that this protein has on other pathologies, such as cancer. It has also been observed that overexpression of NRF2 promotes angiogenesis and resistance to cancer therapies, resulting in poor outcomes^63^. The use of GWAS and computational tools are beneficial for the identification of polymorphic ARE and thus help recognize individuals at high risk of suffering from ROS damage, for whom an antioxidant cocktail could help prevent spaceflight stress and future disease^64^. Thus, more human-based studies are needed in order to cover this important point.

### How ROS affects the physiology of astronauts in space and individuals on Earth

**Fig. 2:**
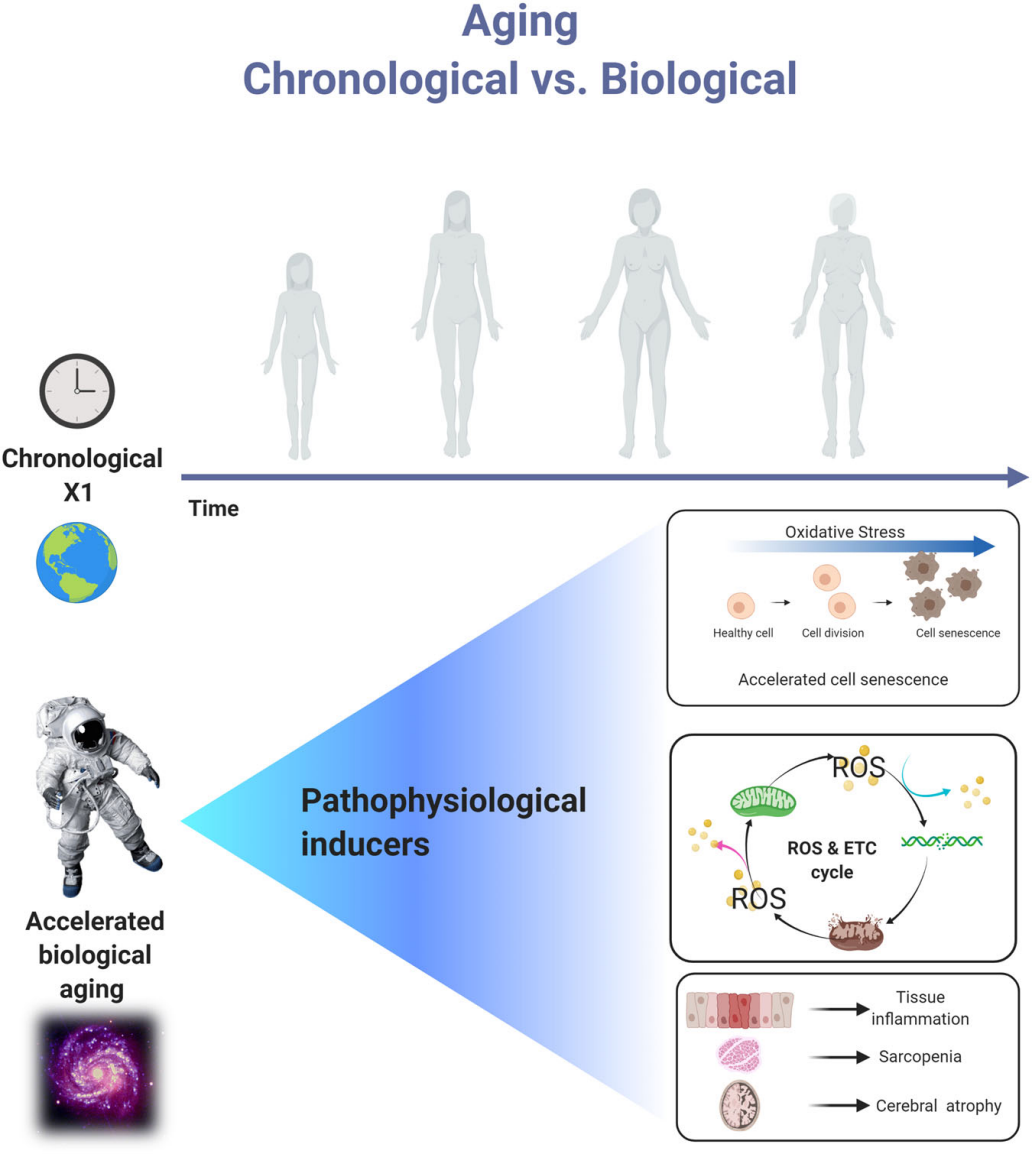
ROS and aging. Biological and chronological aging are usually synchronized. However, space travel may cause an acceleration of biological aging due to pathophysiological stress inducers. Created with BioRender.com.

**Fig. 3:**
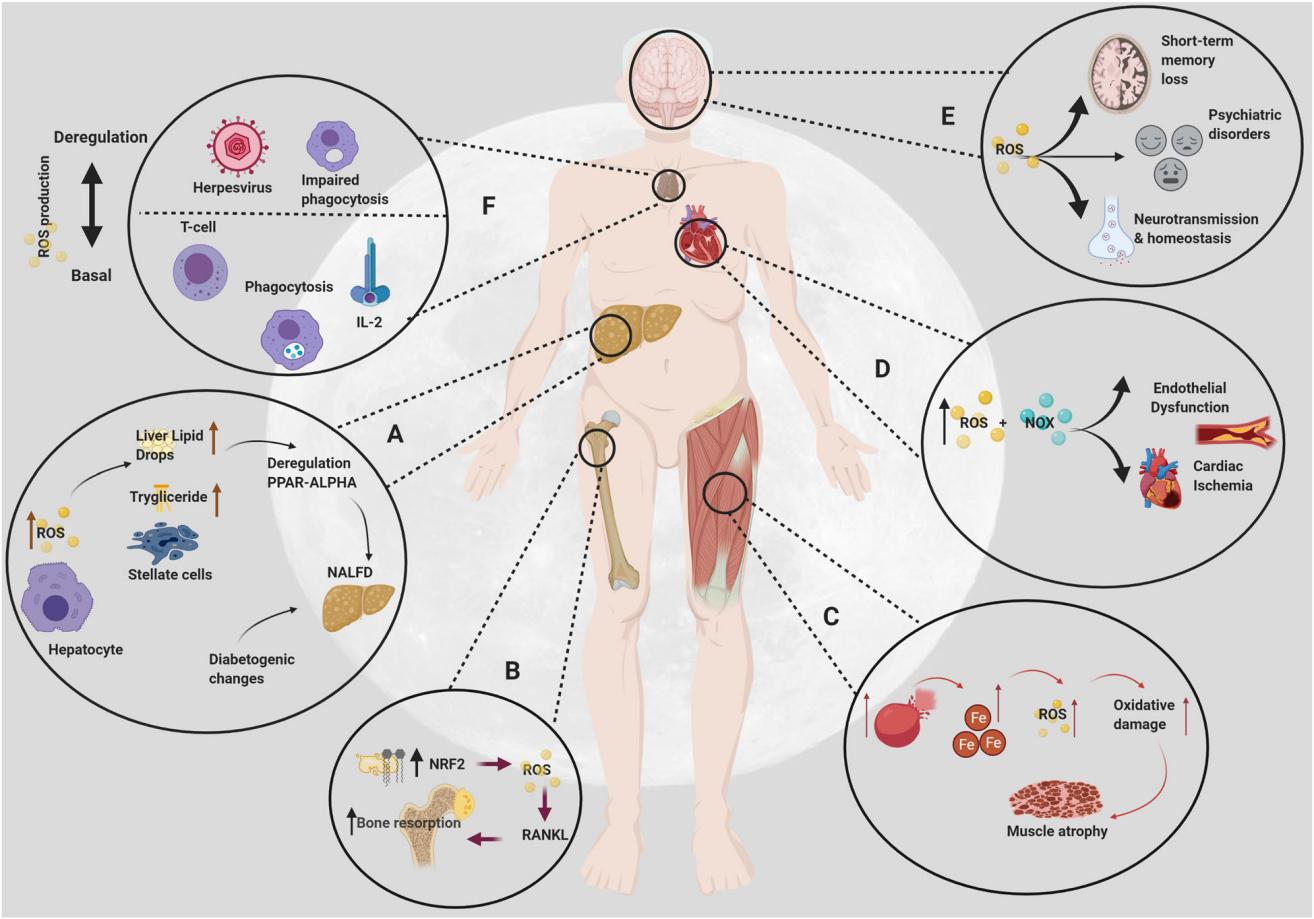
Effects of space missions and ROS on general physiology. **A** High levels of ROS can damage hepatocytes. The damage results in increased lipid droplets in the liver, increased triglycerides, and loss of retinoids from lipid droplets in stellate cells, PPAR-ɑ dysregulation, and diabetic changes that can cause NAFLD. **B** Space radiation damages skeletal lipids and increases the activity of NRF2. ROS acts as a second messenger during RANKL activation and differentiation. This increases bone resorption and osteoclastogenesis. **C** Microgravity and radiation can cause red blood cell destruction, which releases iron. The iron then acts as a cofactor in excess ROS production to accelerate oxidative damage and ultimately cause muscle atrophy. **D** Increased production of ROS and NOX leads to endothelial dysfunction and promotes myocardial necrosis. **E** Oxidative damage causes neurodegeneration that can alter neurotransmitters, induce psychiatric disorders, and dementia. **F** The innate immune system requires the production of ROS in the defense of microorganisms within the phagocytic process and the inflammatory response. However, a dysregulation in the production of ROS induces a lower lymphocyte response, impairing phagocytosis and increasing susceptibility to latent infections such as HSV. Created with BioRender.com.

The psychological stress of living in a confined environment in space as well as the exposure to microgravity and ionizing radiation can increase the harmful effects of ROS on the health of astronauts^65^. It is important to understand how ROS affects biological aging **(**Fig. 2), as well as various organ systems such as the hepatic, musculoskeletal, cardiovascular, neurological, and immunological systems (Fig. 3A–F), in order to develop an antioxidant combination that will prevent an accelerated health loss.

## Aging, ROS, and antioxidants

There are clear differences between biological and chronological aging. Chronological aging refers to the amount of time a person has been alive^66^. It takes into account how human physiology decreases its capacity to maintain homeostasis at a constant rate over time, ultimately leading to death. Oftentimes, chronological age and biological age are synchronized but there can occasionally be discrepancies between the two^66,67^. Unlike chronological aging, biological aging is influenced by pathophysiological inducers, such as ROS, that rapidly accumulate with time in our body and affect the cell’s genome and other intracellular structures, leading to a premature loss of homeostasis^67^ (Fig. 2). Biological aging is related to an increased susceptibility to disease and organ failure mainly due to cellular malfunctions such as genomic instability, reduced stem cell survival, accelerated cellular senescence, perturbed epigenetics, abnormal nutrient absorption, proteostasis, inflammation, and mitochondrial damage^67^. Mitochondrial DNA is very susceptible to damage as mitochondria have fewer repair mechanisms when compared to nuclear DNA. As a result, excess ROS can damage mitochondrial membrane lipids, proteins, and DNA (mtDNA), causing mitochondrial dysfunction^68^. The disturbance of mitochondrial homeostasis causes cell signaling alterations, leading to the activation of cell stress responses toward age-dependent damage^12^.

The NASA Twins Study compared monozygotic twins, one space-bound and one earthbound, over the course of 25 months that spanned preflight, inflight, and postflight points of time^5^. This study reported that, after arriving back to Earth, the space-bound twin had considerably shortened telomeres compared to his brother, an important biomarker in biological aging and associated age-related diseases. These changes in telomere length persisted during the 25 months they were being evaluated and were associated with the stressful events of re-entry back to Earth^5^. Biological aging seems to be controlled by evolutionary conserved pathways involving many factors, including genomic instability, telomere damage, and epigenetic alterations, all of which can be induced endogenously or by exogenous stressors^67^. Mitochondrial dysfunction is linked to excessive ROS production and known to be a consequence of spaceflight missions and a hallmark of diminished genomic instability^5,69^. In vitro and in vivo studies using mice have shown that mice with longer telomeres have higher levels of antioxidants, for example, SOD^70^. Interestingly, previous studies have shown that mice exposed to microgravity for 13.5 days had a decrease in GSH levels and perturbations to their metabolism with a decreased hepatic oxidative defense^71^. Having better endogenous or exogenous antioxidant levels would help mitigate ROS production and maintain telomere length and thus positively influence the health and biological life span of astronauts.

Once this is conceptualized, it is important to emphasize the health burdens that astronauts will face primarily due to increased oxidative stress accumulation, as well other factors such as DNA methylation and genetic inactivation that may lead to accelerated aging^72,73^ (Fig. 2). Understanding the accelerated aging of astronauts in space due to ROS may help us prevent its damaging effects, extend and improve the quality of life of astronauts, and even potentially help those on Earth.

## Hepatic damages and ROS

As we deepen our knowledge of the many physiological and pathological impacts that ROS have on the human body in a spaceflight environment, we must address possible damage to visceral organs as well. The liver in particular is susceptible to ROS damage^71^ (Fig. 3A). Hepatocytes produce ROS as part of the mitochondrial electron transport chain system involved in many metabolic processes^74^. Although not fully understood, mitochondrial dysfunction as a result of ROS damage in hepatocytes may produce pathophysiological changes toward nonalcoholic fatty liver disease (NAFLD), which was observed in mice models tested in microgravity^71^.

It has been observed, using mice as an experimental model, that normal liver gene expression is affected during spaceflight. Interestingly, it was shown that mice aboard the Space Transportation System (STS)-135 had liver damage due to increased liver lipid drops, elevated triglyceride levels, and loss of retinoids from lipid drops of liver stellate cells^75^. All of the aforementioned are associated with dysregulation of the peroxisome proliferator-activated alpha receptor (PPARα)^75,76^. PPARα belongs to a group of nuclear receptor proteins that regulate the expression of genes, especially in the liver, to promote lipogenesis and fatty acid uptake among other metabolic pathways^77,78^. Pro-inflammatory responses negatively regulate PPARα in rodent models of atherosclerosis and early NAFLD that could be affecting astronauts as well^75,76,79,80^. Interestingly, ROS-induced changes have been shown to affect other liver genes related to oxidative defense that, together with mitochondrial dysfunction, may cause accelerated senescence in hepatocytes^71^. In addition to getting a better understanding of how the liver is affected in microgravity conditions, it is also necessary to do more in-depth studies to differentiate the effects of short- and long-term flights in order to generate therapeutic solutions.

## Musculoskeletal damages and ROS

Astronauts experience 12.5% less gravity in space compared to when they are on Earth^81^. The decreased gravitational force and the disuse of muscles accelerate musculoskeletal degradation such as bone loss and muscle atrophy (Fig. 3B, C). Muscle atrophy occurs from the degradation of myofibrils due to both an increase in proteolysis and a decrease in protein synthesis^82^. Astronaut exercise programs have been implemented to reduce these adverse effects; however, they are not always effective. It has been observed that, after only 2 weeks of spaceflight, 20% of muscle mass decreases, while it can decrease up to 30% during longer missions of 3–6 months^83^.

During spaceflight, exposure to microgravity and radiation leads to the destruction of erythrocytes, which release iron bound to hemoglobin that plays a key role in redox mechanisms that are involved in another aspect of muscular atrophy that astronauts experience in space^84^. Iron acts as a cofactor in the production of ROS and hastens oxidative damage that attacks cellular DNA, proteins, and membranes, especially the internal mitochondrial membrane where the electron transport chain reaction takes place^84^. These harmful effects on the musculoskeletal system intensify the loss of muscle mass and muscle strength^84^. These effects of microgravity are not only seen in muscle mass atrophy but also in bone loss attributed to disuse^85^.

Bone loss is a common problem associated with astronauts who return from spaceflight. Changes in ROS seem to be involved in the pathogenesis of bone loss because radiation damages skeletal lipids and the activity of the antioxidant enzyme NRF2 in bone tissue^86^. Additionally, ROS acts as a second messenger during receptor activator of NF-κB ligand (RANKL) differentiation and activation. RANKL-induced ROS production stimulates the activation of the Akt, NF-κB, and extracellular-signal-regulated kinase pathways in osteoclasts, which leads to increased bone resorption activity^87,88^. Nonetheless, many diseases have been linked to oxidative stress, such as osteoporosis. The continued redox state changes act in coordination with all the bone cells: osteoclasts, osteoblasts, and osteocytes. ROS induces apoptosis of osteoblasts and osteocytes, favoring osteoclastogenesis, and the inhibition of mineralization and osteogenesis^89^. Given the effects of changes in the redox state related to the pathogenesis of bone diseases, antioxidants have been associated with increased differentiation of osteoblasts and reduced osteoclastogenesis, which contributes to bone mineralization^89^. Therefore, a strategy that helps balance redox state and antioxidants will promote the treatment and prevention of demineralization.

An astronaut can lose anywhere between 1 and 2% of total muscle mass per month depending on the individuals’ physiology^83^. Because it takes about 48 months of rehabilitation to return to a pre-spaceflight state, physical conditioning and nutrition based on antioxidants may be key to prevent this musculoskeletal damage and reduce recovery time. Interestingly, the use of an antioxidant and anti-inflammatory cocktail consisting of 741 mg of polyphenols, 138 mg of vitamin E, 80 μg of selenium, and 2.1 g of omega-3 did not show to be protective against human hypoactivity induced by skeletal muscle deconditioning by the head-down bed rest model^54^. The presence of antioxidant molecules accentuate skeletal muscle waste pointing that physiological amounts of ROS and reactive nitrogen species (RNS) could be key to triggering a positive adaptation that could compensate for part of the damages observed in the control group^54^. New antioxidant combinations at different doses should be investigated to control the complex redox mechanisms involved in muscle damage.

## Cardiovascular damage and ROS

The cardiovascular system is made up of the heart, the blood vessels (arteries, arterioles, veins, venules, capillaries), and the blood that is responsible for oxygenation of the tissues. This system can also be dysregulated by excessive ROS production especially during spaceflight^65^ (Fig. 3D). One of the most affected sites is endothelial tissue, which plays an essential role in cardiovascular homeostasis by regulating blood fluidity and fibrinolysis, vascular tone, angiogenesis, leukocyte adhesion, and platelet aggregation^90^.

Under normal physiological conditions, ROS regulates heart development, cardiomyocyte differentiation, vascular tone, and excitation contraction coupling^91^. Under unregulated conditions, elevated ROS and NADPH oxidase (NOX) levels have been implicated in cardiac hypertrophy, heart failure, cardiac ischemia–reperfusion injury, and diabetic cardiomyopathy^91^. The enzymes involved in the process of generation of ROS are NOX, xanthine oxidase (XO), lipoxygenase, NOS, and myeloperoxidase and certain NOX subtypes, such as NOX1 (vascular smooth muscle cells), NOX2 (endothelium, vascular smooth muscle cells, adventitia, and cardiomyocytes), NOX4 (endothelium, vascular smooth muscle cells, cardiomyocytes, and cardiac stem cells), and NOX5 (vascular smooth muscle cells)^65^. NOX participates at the cardiovascular level in angiogenesis and blood pressure regulation as well as the development of diseases like hypertension, left ventricular hypertrophy, and myocardial infarction^65^. The end result of this ROS generation is endothelial dysfunction caused by oxidative stress, which produces more ROS and less NO. When there is less NO production, there is greater endothelial damage, which leads to the previously mentioned cardiovascular disorders^65^. Therefore, understanding ROS and NOX production in the cardiovascular system during space missions is essential.

Several in vitro and in vivo studies have been conducted related to ROS production, exposure to microgravity, and radiation. One of the studies was performed in rats using the bed rest and hindlimb unloading (HLU) model, which revealed that after 3 weeks of exposure to simulated microgravity there is an increase in O_2_^−^ and O_2_ levels in cerebral and carotid arteries^92^. A study of human endothelial cells from the umbilical vein sent on a 10-day spaceflight showed that 1023 genes were affected. These genes are involved in cell adhesion, oxidative phosphorylation, cell cycle, stress response, and apoptosis. Among the proteins that were encoded with the highest overexpression was the pro-oxidative protein Trx^93^. Finally, another study using high-energy and high-atomic number (HZE) iron ions as a model of space radiation to rat bodies generated different conclusions. It was shown that there is an increase in aortic stiffness and tension, two characteristics that coincide with the existence of endothelial dysfunction^94^.

## Neurological damage and ROS

The tissue of the nervous system is made up of mainly two types of cells: the neurons that are the functional units of the system, and the neuroglial cells that are the supporting cells. These human brain cells have a high metabolic demand, so they are remarkably susceptible to the detrimental effects of ROS. Nevertheless, adequate ROS concentration regulates neuron activation to allow synaptic connection^95^. Oxidative damage indiscriminately alters neurological signaling that regulates many physiological processes such as neurotransmission, homeostasis, and degeneration^96^. It is important to note that one of the most important damages observed during spaceflight are those related to brain function (Fig. 3E).

Scientific evidence of the neurological damage caused by ROS has been gathered in animal models and in vitro models. A study conducted in rat PC12 cells reported that exposure to simulated microgravity contributes to oxidative damage. This article showed a reduction of antioxidant enzymes (SOD, GPx, and CAT) and increased ROS production in these neuronal cells 4 days after its onset^97^. Another study that used *Drosophila melanogaster* showed a significant reduction of dopaminergic neurons, compared with the control group, under hypergravity conditions in a similar way to human’s Parkinson’s disease (PD)^98^. Additional genetic studies mention a correlation between ROS not only with PD but also with Alzheimer’s disease, cerebral ischemia, multiple sclerosis, and neurodegenerative and depressive disorders^99^. It has been found that damages caused by oxidative stress in the mitochondria are associated or responsible for diseases, such as schizophrenia, autism, depression, and anxiety^99^. This may be attributed to enhanced signaling levels of GMP-PKG that increase the concentration of phosphodiesterase 2 and NOX2-dependent mechanisms that are correlated with the aforementioned diseases and ROS, especially in the pathogenesis of psychiatric disorders^99^. Additionally, a defect in the electron transport chain has been reported to be related to autism and schizophrenia^99^.

As space travel technology improves, we must mitigate the plethora of neurological pathologies associated with a possible increase in ROS production during extended space missions. We must also remedy other neurological damages caused by spaceflight such as neuro-ocular syndrome, which occurs in approximately 40% of astronauts due to brain fluid displacement and associated adaptations to the hostile environment^5^.

## Immunological damage and ROS

Microgravity and ionizing radiation are stressors that alter the immune system of astronauts during space missions^24^ (Fig. 3F). These stressors induce dysregulated production of ROS, which disrupts cellular signaling, such as migration, in the immune system cells^100^. Additionally, these hostile spaceflight environments impair lymphopoiesis in lymphoid organs, mainly the thymus, impairing the acquired immune system even further and increasing susceptibility to infection^101^.

ROS have different functional roles in immunologic cell signaling. In the innate immune system, ROS are key elements in both the coordination of migration of polymorphonuclear leukocytes to sites of injury as well as the activation of inflammasomes^100^. Inflammasomes activate Caspase-1, interleukin (IL)-1β, and IL-18 to produce pyroptosis that releases damage-associated molecular patterns (DAMPs) to subsequently recruit more immune cells to mount a robust immune attack against pathogens^102^. Recurrent infections like pneumonia, abscesses, and osteomyelitis are primarily seen in patients with impaired ROS-generating phagocytes, as ROS production is necessary for neutrophils and macrophages to effectively phagocytose bacteria^30^. In the adaptive immune system, ROS is a critical second messenger for T cell receptor signaling and T cell activation. They induce the production of IL-2, which is critical for CD4+ and CD8+ T cell proliferation^103^, both of which are important for destroying invading microorganisms.

Studies performed on mice during a 1-month space mission aboard the ISS revealed that microgravity caused atrophy of the murine thymus^104^. Given that the thymus is a primary lymphopoietic organ, T cell differentiation and proliferation were reduced^104^. Likewise, while in space, astronauts experience fluctuations of gravity that can cause dysregulation of CD8+ T cell infiltration in ganglions. This dysregulation can permit reactivation and/or shedding of latent viruses like herpes simplex virus (HSV), varicella zoster virus, Epstein–Barr virus, and human cytomegalovirus^101^ that would otherwise be easily kept at bay by a functional adaptive immune system.

All in all, space is a hostile environment that also disrupts normal immune cell functioning. We should address this, given that an impaired ability to activate the immune system leaves astronauts unable to produce an effective response against pathogens. Interestingly, endogenous antioxidants like GPx and exogenous antioxidants like vitamin C, vitamin E, and β-carotene mitigate immunological ROS damage and thus may be key to maintaining a sturdy immune system in space^14^.

### Antioxidant administration

The human body has been shown to interact in a different way pharmacodynamically and pharmacokinetically in space as compared to Earth, resulting in a lack of effectiveness or even cytotoxicity when metabolizing drugs during spaceflight missions^105^. It was reported in 1999 that 94% of astronauts in space shuttle missions were taking some sort of medication: 47% were taking drugs for space motion sickness; 45% for sleep disturbances; and 3% for headache, backache, or sinus congestion^106^. This study showed that 80% of the drug-dose events were perceived as effective by the recipients^106^. Additionally, inflight studies of orally administered medications showed that doses were not as effective as expected. For example, acetaminophen taken orally showed a significant difference between preflight and inflight salivary levels, especially in the absorption rate^105,107^. As a result, this phenomenon could alter the pharmacokinetics and pharmacodynamics of antioxidants by changing absorptivity^108^. Delayed gastric emptying due to microgravity conditions or the side effects of medications being administered to control nausea induced by spaceflight could alter the absorption of antioxidant compounds in the gastrointestinal tract^109^. The reduction of total body water due to fluid displacement and renal excretion can also affect the volume of distribution of the drugs and antioxidants consumed^109^. When an astronaut is in orbit, a fluid shift from the intracellular to the extracellular compartment occurs due to increased permeability of capillary membranes^110,111^. However, after a few days, diuresis reduces the extracellular fluid and plasma volume^110^. This absence of a hydrostatic pressure gradient can diminish the baroreceptor response^112^. This variation in blood and plasma flow can affect the rate of drug absorption, distribution, and elimination^110,113^. Therefore, effective doses of antioxidant compounds could vary in space and should be further studied.

Cardiac arrhythmia is a problem that has been reported in long-duration spaceflight, especially related to a QT prolongation observed on electrocardiograms^110,114^. Arrhythmias could be caused by mitochondrial oxidative stress; however, antioxidants such as resveratrol (RES) have been shown to reduce this problem in vivo^115,116^. QT prolongation is a delay in ventricular repolarization of the heart muscle. It is important to consider this because some drugs used during spaceflight may cause a delay in the QT interval and thus could increase the risk of arrhythmia^114^. Cardiac muscle loss can affect how drugs bind to heart tissue, thus altering absorption^105^. Although this mechanism is not well understood, spaceflight may be associated with hypersensitive reactions to some medications^110,117^. Drugs and antioxidant compounds may have an effect on cardiac arrhythmias that need to be further explored as they could bring great benefits for the cardiovascular health of astronauts during spaceflight.

Currently, few studies have been conducted taking into account the pharmacokinetics and pharmacodynamics of both antioxidants and other drugs. Furthermore, the studies that have done so had a small sample size and did not have a consistent methodology^108^. Finally, the parameters related to drug research are only partially described^108^. Unfortunately, there is no evidence about pharmacogenetics in space^108^. This field is important to future research because the drug implications may not be the same for all astronauts, which can vary depending on factors such as ethnicity and age.

More assays need to be done to investigate the different effects that drugs and antioxidants have on Earth versus in space. These assays could be performed on Earth by simulating microgravity conditions and observing the resulting physiological changes in various situations. The HLU models are affordable and viable alternatives to explore the differences in pharmacokinetics and pharmacodynamics. However, many spaceflight conditions cannot be replicated and some changes in space may not be consistent with Earth models^108^. The understanding of the changes in pharmacokinetics and pharmacodynamics in space would help determine the best dosage and results of a drug when space travel becomes available to the public.

Based on an analysis of dosage (dose and frequency of dose) used by U.S. crewmembers on the ISS, we propose that monitoring levels of endogenous antioxidant such as SOD, GPx, GSH1 could be performed every 7 days and may help to understand how well the antioxidant response in astronauts is and the need to modify supplementation. Analyzing and establishing dosage for different antioxidant combinations can allow us to determine the effectiveness of a particular combination and to reduce excessive supplementation^118^. It is our opinion that antioxidants should be taken regularly during spaceflight, more than a single or sporadic dose, especially during long-term missions.

Even if direct inflight analysis of antioxidant levels in body fluids could be useful, they are difficult to perform due to the lack of equipment and reagents on board of spaceships and ISS^119^. In the NASA twins study, most of the analysis was performed on Earth with preserved samples taken from one of the twins; this is a recurrent practice that allow to understand any particular inflight risks; however, it limits the capacity of response on site^5,119^. Easy to perform analyses are currently under development or being tested for amplifying and sequencing DNA, measuring the levels of transcripts, proteins, and metabolites that may help to establish routine evaluations of the astronauts health, antioxidant levels, or cellular stress during fights, to take effective preventive measures against disease^119^.

The available literature suggests that exogenous antioxidants protect organisms from oxidative stress to varying degrees. For example, a 2011 study administered RES to rats as a nutritional countermeasure to ROS damage^120^. The study showed that RES helped to maintain the capacity of mitochondria to oxidize palmitoyl-carnitine and reversed the decreased GSH versus glutathione disulfide ratio, a biomarker of oxidative stress^120^. Nevertheless, it is unlikely that a single exogenous antioxidant is strong enough to reverse the wide range of negative effects induced by oxidative stress; even supranormal doses could be considered^35^. Interestingly, some antioxidants may have oxidative effects when used in combination with other nutrients^14^. The alpha-tocopherol, β-carotene cancer prevention study in Finland showed that both antioxidants did not reduce the risk of lung cancer in male smokers with an age between 50 and 69 years. This study observed a higher incidence of lung cancer in the group receiving β-carotene. However, authors recommended more research and careful examination of the results^121^. Carotenoid activity has been generally described as antioxidant; however, depending on the study and dosage they can act as pro-oxidants. Carotenoids have shown to have antioxidant activity when applied in combination with vitamin C and tocopherol, but this activity seems to be affected by the oxygen concentration outside the cell. At a low oxygen pressure, carotenoids function as chain-breaking antioxidants. In contrast, at high oxygen pressure they exhibit pro-oxidant activity in in vitro studies^122^. In vitro, in vivo, and clinical assays are needed to improve the understanding of the antioxidant or pro-oxidant activity of molecules such as carotenoids, the identification of the oxidation products formed, and the physiological significance of the results is needed to establish dose and combinations, which may change when astronauts go to space^122^. Additionally, it has been shown that the dosage of antioxidants administered with the aim of treating deficits or maintaining the normal physiological processes are different and depend on the molecule. Higher doses of molecules such as vitamin C could be administered and be beneficial to patients with high oxidative stress such as in the cases of burns, severe trauma, and critical illness^123^. Therefore, a combination of exogenous antioxidants, in the form of a cocktail, could provide synergistic and therapeutic effects in response to oxidative stress on the human body while in space. However, the current key points are necessary to develop safe and effective antioxidant combinations and doses^124^.

### Characteristics and evidence of the effectiveness in the use of antioxidants and their possible administration during spaceflight

In this section, we present characteristics and examples of different dosages of common antioxidants based on biomedical literature and scientific publications regarding its use in a clinical scenario or based on the recommendations that resulted from each study. These doses may differ from the recommended dietary allowances (RDA) as they are applied in a particular research context or to mitigate a clinical deficiency and in the case of disease. The RDA values of antioxidants are based on an adjusted estimation of the average physiological requirement of a nutrient, bioavailability, and variation of their intake by the U.S. population; doses of antioxidants may be higher to optimize biological processes such as immune protection; however, more research is needed to determine the best dose in each scenario^125–127^. As authors, we recommend that the following doses should be taken only as a reference in the context of this article, which suggest the development of an antioxidant cocktail that may prevent ROS damage in astronauts during missions or space travel, an accredited physician or space agency specialist should be consulted before taking any of the antioxidants or modify the RDA recommendations.

Exogenous antioxidants such as vitamin C, vitamin B complex, vitamin E, B-carotene-vitamin A, L-SeMet, RES, coenzyme Q10, and melatonin still need more validation in clinical trials. Their application to a more specific set of patients with the knowledge of their genetic background is important to understand the efficacy of antioxidant combinations. Applying the “Right species, Right place, Right time, Right level, and Right target” set to patients could help to improve the understanding of antioxidant effects on Earth^15,126,128–131^. Astronauts are constantly exposed to radiation, which accumulates with time, during spaceflight missions^132^. The main operational countermeasure is to limit astronaut exposure by shortening the mission duration on the ISS to 3–6 months^132^. Future long-term missions, such as going to Mars, could last 2 years and strategies to prevent the damages of excess ROS production due to radiation are important. As each vitamin and antioxidant may lead to prevent or decrease ROS damage in cells, it is relevant to mention their main characteristics and evidence of their use in the prevention of diseases for future research and application in space exploration. The clinical benefit or the prevention of disease of each of the following antioxidants should be studied further to provide evidence based on quality systematic reviews to support any recommendations for their use on Earth or during space travel.

### Vitamin C

Vitamin C, also known as ascorbic acid, is an essential antioxidant in humans and animals^133^. However, due to a genetic mutation of the L-gulonolactone oxidase gene in the last step of the synthesis pathway, humans have lost the ability to make vitamin C and thus must obtain it from the diet^134^. The metabolite is found in many fruits and vegetables, especially in citrus fruits^134^. As an important reducing agent in the body, catalyzing key enzymatic reactions, and the synthesis of collagen, vitamin C deficiency can result in detrimental health effects, such as scurvy^133^. Vitamin C has been used to treat diseases or minimize their negative effects on health. Supplementary Table 1 provides information regarding vitamin C doses and disease treated. In Supplementary Table 2, we show the evidence of the use of vitamin C to prevent or treat diseases related to the cardiac, respiratory system, immunological to aging.

Ascorbic acid could be of use during spaceflight where its different beneficial properties to our metabolism that, specifically related to immune function, could be exploited. It is important that astronauts are able to keep a normal immune response during spaceflight, as the immune system is particularly vulnerable to oxidative attack. ROS generated during the activation of immune cells can be scavenged by non-enzymatic antioxidants, such as vitamin C. Studies have shown that this exogenous antioxidant is highly concentrated in leukocytes^135^. It works as a regulator of redox and metabolic checkpoints, controlling the activation and survival of immune cells^136^. This immunomodulating effect of vitamin C may occur through the inhibition of proinflammatory cytokines from NF-κB or by hampering T cell apoptosis-signaling pathways^135,137^.

Vitamin C has other antioxidant effects, in addition to its ability to enhance immune function. For example, it is a cofactor for eight human enzymes, including hydroxylases needed for collagen synthesis^133,136^. As vitamin C decreases the expression of proinflammatory mediators, it has important anti-inflammatory properties, especially during infection and wound healing^138^. It has also been shown that ascorbic acid can act as a protective factor for the skin against environmental oxidative stressors^139^. The protective effect of this vitamin against certain toxicity is linked to the increase in formation of the 4-hydroxy-2(E)-nonenal-glutathione conjugate and its phase I metabolites^140^. A summary of the evidence of its use are shown in Supplementary Tables 1 and 2.

### Vitamin E

Vitamin E is a group of essential lipid-soluble compounds that can be found in foods such as nuts, seeds, leafy vegetables, vegetable oils, and fortified cereals^141^. It is stored within the fatty tissues of animals so it does not need to be eaten everyday^141^. Vitamin E has anti-inflammatory and antioxidant properties and plays an important role in innate and adaptive immune cell signaling^142^, which indicates potential for space mission application. Supplementary Table 3 provides information regarding vitamin E doses and disease treated. In Supplementary Table 4, we show the evidence and results of the use of vitamin E in randomized clinical trials (RCTs) and other research papers.

Vitamin E works as an antioxidant by conferring protection against lipid peroxidation, which produces metabolites associated with oxidative stress^143^. This compound when combined with selenium may reduce ROS formation in tissues that would otherwise damage lipid membranes^142^. Similarly, when combined with GSH and vitamin C, they are able to synergistically scavenge lipid soluble-free radicals^142^. On Earth, the benefits of vitamin E are related to the prevention of chronic diseases linked with oxidative stress^136^. It has been observed that y-tocopherol levels in plasma decreased by 50% after spending 4–6 months in space, while α-tocopherol levels remained stable^142^. As such, regulating levels of this antioxidant within our astronauts may be important.

Additionally, the intake of this vitamin has positive effects on the human immune system. It increases the production of IL-2, lymphocyte proliferation, natural killer cell activity, and the resistance against different infectious agents by promoting T helper type 1 (TH1) response and decreasing TH2 response^139^. This enhances the immune response by supporting macrophage and monocyte mediation^135,139^. It also improves T cell functioning by lowering production of Prostaglandin E2^142^. Furthermore, it has anti-inflammatory properties through the inhibition of the NF-κB pathway^136^.

Further research is needed to better understand the pharmacokinetics and pharmacodynamics of vitamin E in space. The administration of exogenous vitamin E could have potential benefits in preventing inflammatory processes and infections/disease processes as shown in Supplementary Table 3. Studies have shown that it can confer positive health benefits against ROS oxidation and promotion of a strong immune system. However, as with all vitamins and nutrients, it is important to not surpass recommended doses, as high concentrations of vitamin E have been shown to be deleterious for health^144^.

### Vitamin A

Vitamin A, also known as retinol, is a vital micronutrient for multiple processes in the human body, including sight in the retina and antioxidation by retinoic acids. However, high doses of vitamin A have detrimental effects and can be lethal. The administration of β-carotene as a precursor of vitamin A prevents the accumulation of pure exogenous vitamin A and its toxic effects^145–147^. β-Carotene is a natural orange pigment with important antioxidant properties that is part of the human diet^148^. There are two main isomers of beta carotene, part of the retinoic acids: all-*trans-*β-carotene, which is more abundant and is the most suitable precursor of vitamin A, and *cis-isomers* like 9*-cis*-β-carotene, which is a result of exposure to light^149^. For structural reasons and based experimental data, β-carotene works as a radical scavenger, especially with peroxyl radicals, and prevents the formation of singlet oxygen^146^.

Studies have shown synergism between beta carotene, ascorbic acid, and alpha tocopherol by the formation of the ascorbyl radical^150^. These mechanisms were observed in a 90-day follow-up study with 20 athletes who were administered an antioxidant cocktail that contained high concentrations of β-carotene without any change in vitamin A concentrations^147^. Furthermore, a clinical trial showed that a group given a cocktail of β-carotene, vitamin E, and vitamin C had 1.2–1.6 times more antioxidant activity than the placebo group^147^. Since beta carotene is an important antioxidant, and it can enhance its activity with fellow antioxidants, it is important to study the different interaction between beta carotene and ROS within the various organ systems and how that correlates to long-term spaceflight.

Increased oxidative stress during spaceflight can affect the cardiovascular system and increase the risk of cancer^142^. Vitamin A and β carotene serve as antioxidants to reduce the risk of cancer and coronary heart disease^142^. In Supplementary Table 5, we provide information regarding β-carotene doses and disease treated in RCT and other research papers. Vitamin A may play a critical role in maintaining antioxidant health during spaceflight^142^. The data provided by a NASA study of cataracts and nutritional intake revealed that β carotene had a protective effect for some types of cataracts in astronauts^151^. A recent meta-analysis provided similar results supporting an inverse association of a carotene (with vitamin E, lutein, and zeaxanthin) with age-related cataract^152^. In addition, there is a significant difference of serum level retinol between pre- and post-flight samples but not during flight. Serum retinol decreased from 0.73 ± 0.17 to 0.59 ± 0.13 µg/mL on landings occurred in Russia and increased from 0.52 ± 0.09 to 0.63 ± 0.12 µg/mL on landings in the US^48,142^.

### L-selenomethionine

L-SeMet is the amino acid methionine with the sulfur being replaced by the trace element selenium^153^. Methionine is an essential amino acid, and selenium is an antioxidant with ROS scavenging properties. L-SeMet has a fundamental role in the protection of tissues from lipid peroxidase damage, which could target cell membranes^154^. Studies have shown that a 5 μM L-SeMet supplement in the medium of human thyroid epithelial cells (HTori-3 cells) may have an important role in the fight against space radiation, especially in regard to HZE. These particles can increase cytotoxicity and oxidative stress and decrease the total antioxidant status^155–157^. Studies have proposed that these negative effects are preventable by using L-SeMet, suggesting that this exogenous antioxidant is a useful tool to countermeasure HZE particles^158^.

Furthermore, it has been observed that the usage of L-SeMet in a culture of human thyroid epithelial cells upregulates the gene expression of ATR and CHK2^157^. ATR is an important component of the DNA damage response pathway and CHK2 is a cell cycle checkpoint regulator and tumor suppressor^157^. Defects in these genes contribute to the development of hereditary and sporadic cancer^157^. However, supplementation with L-SeMet might enhance ATR and CHK2 expression and might prevent cancer caused by HZE particle radiation exposure^157^. Moreover, selenium concentrations have been observed to decrease by >10% after spaceflight missions^142^. Selenium is a cofactor of multiple enzymes (e.g., GPx, TrxR) that have strong antioxidant activity. GPx plays an important role in the protection of cells against RNS and ROS. TrxR is a substrate used to maintain the Trx/TrxR system in a reduced state by removing hydrogen peroxide^154^. Deficiency of selenium can lead to immune dysfunction, Keshan disease, and even death^142^. It has also been shown that L-SeMet and selenium have potential effects in the prevention of heart diseases, cancer, and immune system-related diseases^159^.

### Resveratrol

RES is a polyphenol compound found in foods like grape skin, blueberries, and red wine^160^. It has potent antioxidant/free-radical scavenging, anti-inflammatory, and anti-diabetic properties that play a key role against oxidative stress, apoptosis, inflammation, and mitochondrial dysfunction^161^. In Supplementary Table 7, we provide information regarding RES doses and disease treated in RCT and other research papers. A recent study concluded that RES has the potential to prevent musculoskeletal deconditioning that occurs during mechanical unloading in partial gravity conditions that astronauts could be exposed to^160^.

The potent oxygen-scavenging property that RES has is attributed to its three hydroxyl functional group molecular structure: it works as an antioxidant by promoting nitric oxide (NO) production, suppressing platelet aggregation, and enhancing high-density lipoprotein cholesterol^161,162^. In astroglial cells, RES prevents the depletion of important antioxidants, like GSH, GPx, SOD, and HO-1^161,163^ whose decrease triggers inflammation and oxidative stress^161,163^. In lipopolysaccharide (LPS)-stimulated murine macrophages, RES downregulated the expression of inflammatory cascade markers, IL-6 and tumor necrosis factor (TNF)-alpha^161,164^. Additionally, RES attenuates the cytotoxicity of H_2_O_2_ that is capable of damaging mitochondria^165^. Studies showed that H_2_O_2_ led to mitochondrial dysfunction, which was associated with decreased ATP levels, a lower mitochondrial membrane potential, and an increase in intracellular ROS production^165^. Furthermore, RES has been linked with autophagy activation and protection against mitochondrial dysfunction^165^.

Interestingly, RES has also been associated with the preservation of muscle health, which usually suffers in extraterrestrial conditions like a Martian gravity analog^160^. Research has shown that RES has hypoglycemic effects, which may indicate that it plays an important role in glucose and energy homeostasis in muscle tissue by regulating insulin sensitivity. It is important to note that RES supplementation before, during, and after mechanical unloading enhances insulin sensitivity as well as muscle recovery^160^. The administration of RES mitigates muscle atrophy, preserves muscle mass, maintains bone, and promotes muscle recovery in astronauts^160^. Though RES is a relatively new compound that is being studied, it contains many promising properties that may be beneficial to astronauts during space missions as they explore the infinite.

### Isorhamnetin and luteolin

Isorhamnetin and luteolin are flavonoids that have been used in traditional Chinese medicines due to their antioxidant properties^166,167^. Flavonoids are a class of natural compounds that can have beneficial effects due to the radical scavenging properties of their hydroxyl groups^167,168^. In Supplementary Tables 8 and 9, we provide information regarding the use of isorhamnetin and luteolin doses and diseases treated in RCT and other research papers. In one study with 20 flavonoids under simulated microgravity, isorhamnetin and luteolin showed preventative qualities in regard to oxidative stress in neuroblastoma-derived cells (SH-SY5Y)^169^. Clinostat, an experimental device that uses rotation to negate the effects of gravitational pull, was used to simulate microgravity^169^. The study found that both isorhamnetin and luteolin protected SH-SY5Y cells by preventing the increase of ROS, NO, and 3-nitrotyrosine levels and the decrease of antioxidant power induced by microgravity^169^. Additionally, treatment with isorhamnetin and luteolin reduced the expression of inducible nitric oxide synthase, a fundamental enzyme for catalyzing NO production^169^. This suggests that isorhamnetin and luteolin might protect against oxidative stress in space through ROS-NO pathway modulation^169^.

As a major derivative of quercetin, the most commonly found flavonoid in edible plants, isorhamnetin is known for its antioxidative and anti-proliferative effects^170^. In addition, a 2013 study investigated the anti-inflammatory activity of isorhamnetin^170^. This study found that isorhamnetin decreased the amount of cells with cyclooxygenase-2 (COX-2) in rats with carrageenan-induced paw edema^170^. As ROS production can lead to COX-2 induction, the decrease of COX-2 suppressed the production of LPS-induced ROS and reduced apoptosis^170^. As a result, the oxidative damage likely decreases with isorhamnetin supplementation^170^. Furthermore, the study reported that isorhamnetin increased the nuclear translocation of NRF2 and consistently increased antioxidant response element reported activity and protein levels of HO-1 and glutamate cysteine^171^. NRF2 dissociates from Keap1 and induces the expression of HO-1, which has antioxidant response elements located in the promoter region^170,172^. This mechanism has been found to reduce the production of ROS and might contribute to the downregulation of COX-2^170,172^. Thus, isorhamnetin may decrease oxidative damage in cells and be a good candidate for the antioxidant cocktail given to astronauts.

It has been demonstrated that luteolin has both anti-inflammatory and antioxidant effects, similar to isorhamnetin. Oral administration of bleomycin-instilled C57BL/6J mice has shown to suppress neutrophil infiltration and the increments of TNF-alpha and IL-6^173,174^. In a study using mice with cerulein-induced severe acute pancreatitis, luteolin suppressed the increase of TNF-alpha and IL-6 levels in the pancreas and serum^173^. Luteolin also suppressed the increase of malondialdehyde and the enhancement of SOD1 in the pancreas^173^. No liver and kidney toxicity was reported^173^. In addition, the results showed decreased NF-κB activity, reduced lipid peroxidation, and increased IL-10 levels^173^. This effect depends on HO-1 induction^175^.

### Coenzyme Q 10 (CoQ10)

CoQ10, also known as ubiquinone, is a key antioxidant for cell function that decreases ROS production. It is a fat-soluble micronutrient synthesized by all human cells and tissues that can also be taken exogenously to mitigate ROS damage, as CoQ10 is able to reduce superoxide production in mitochondria^176,177^. CoQ10 has an important role in the mitochondrial electron transport chain, as it accepts and transfers electrons between complexes I–III^178^. Additionally, CoQ10 participates in a variety of functions inside the cell. CoQ10 is able to prevent damage to DNA and proteins, lipid peroxidation, stabilize calcium channels to stop their overload, activate the uncoupling proteins for heat generation, increase cyclic adenosine monophosphate (cAMP) levels, and enhance the activity of SIRT1 and PGC-1α, which have important roles in improving mitochondrial biogenesis and function^176,179–181^. Numerous studies have shown that the intake of exogenous CoQ10 could help to prevent the progression of illness, such as heart failure, metabolic syndrome, NAFLD, diabetes mellitus, and several neurodegenerative diseases^177,181–186^. However, more evidence is needed before its application and recommendation in clinical practice. In Supplementary Table 10, we provide information regarding the use of CoQ10 in RCT and other research papers.

CoQ10 is produced by the mevalonate cycle in healthy individuals^177,181^. Two-to-5 mg/day can also be obtained from foods, such as dark vegetables, soy, meat, and fish^181^. In humans, CoQ10 could be administered once or twice per day with 100–200 mg doses, with 22–400 mg/day considered as the safe range. When administered exogenously at these concentrations, benefits in lipid metabolism and metabolic syndrome have been observed^177,181^. CoQ10 is absorbed in the small intestine, transported by chylomicrons to the liver, then incorporated into low-density lipoproteins for its distribution to various organs, including the spleen, adrenal glands, and heart^177,181^. CoQ10 elimination occurs through the bile ducts and feces with a small fraction excreted in urine^181^. Interestingly, CoQ10 levels after oral administration can increase between 1 and 2 h circulating in plasma in the reduced ubiquinol form^181,187,188^. The maximum concentration of CoQ10 is reached within 6–8 h with a half-life of 34 h^177,181–188^.

CoQ10 inhibits lipid, protein, and nucleic acid ROS oxidation, as well as apoptosis in cells such as corneal keratinocytes that are exposed to radiation in vitro and in vivo^189,190^. These effects are of great interest for space agencies such as the Agenzia Spaziale Italiana and NASA given that they need to prevent or minimize the effects of radiation and microgravity in the nervous system and sensory organs^189,190^. Interestingly, the international collaboration between both agencies has shown that the use of CoQ10 prevents simulated microgravity-induced apoptosis and lowers the accumulation of DNA damage foci together with telomere dysfunction^189^. The increase of ROS production due to the exposure to space radiation and microgravity could be associated with premature aging and loss of biological fitness in astronauts^191,192^. Interestingly, in the NASA twins study, the twin that stayed on board the ISS for 1 year showed lengthened telomeres. However, after he returned to Earth the telomere length shortened very rapidly, surpassing the number of shortened telomeres in the twin who stayed on Earth^5^. CoQ10 levels decrease with age and are correlated with the development of chronic diseases and excess ROS production^177,193^. CoQ10 supplementation would provide astronauts with protection from the excess ROS produced by their cells and even from the possibility of accelerated aging^179,189,194^. The use of CoQ10 should be further studied with focus on dosage in space and its effectiveness in preventing ROS-related damage in the organs of astronauts.

### Workflow proposal for assessing astronaut’s resistance to ROS damage, infight monitoring of ROS production, cellular stress, and a personalized preventive and inflight ROS protection based on antioxidant cocktail supplementation

Previously, we mentioned that the direct evaluation of endogenous or exogenous antioxidant levels are not routinely performed as part of astronauts’ health tests to our knowledge. However, the study of markers of ROS damage have been done with research purposes, observing an increase in astronauts^48,50–52^. NASA observed astronaut twins and found an increase in inflammatory markers and abnormal gene expression patterns in processes related to the metabolism of ROS and mitochondrial function compared to the twin on Earth^5^. Providing inflight information to the crew and on land control centers about the levels of ROS production and the levels of antioxidant endogenous protection in astronauts could help to identify critical points during space missions where special care should be established in order to maintain the health of the crew.

The analysis of ROS, oxidative damage, antioxidant levels, and other biomarkers of health based on liquid biopsies could have been hampered by the lack of equipment and reagents available on spaceships and ISS; however, novel technologies are being applied to analyze a wide range of biological markers^119^. New generation of automated and miniaturized machines that use microfluidic technology, on-chip DNA amplification, and reverse transcriptase–quantitative polymerase chain reaction (qPCR) could provide the on-board capacity to measure ROS and endogenous antioxidant production by routine mRNA analysis^119^. With suitable equipment, we propose to estimate ROS levels in cells, such as in peripheral blood mononuclear cells, by using fluorogenic probes and flow cytometry^49,119^. Oxidative damage estimation could be performed by the estimation of advanced oxidation protein products, lipid peroxidation, and 8OHdG in the plasma or serum of astronauts^49^.

New and direct markers of cellular damage at an organism level could be used to evaluate astronauts’ environmental or psychological stress. The estimation of circulating-cell-free mitochondrial DNA (ccf-mtDNA) by qPCR in blood plasma or serum is a novel biomarker under development of physiological damage, easy to access, and of great biological significance. Cells under stress can liberate vesicles containing mitochondria as danger signals or as a way to exchange material aid other cells in danger; this property has been observed in mesenchymal stem cells^195–198^. Assessing the presence of extracellular mitochondria or their fragments by measuring ccf-mtDNA levels could allow on-flight direct estimation of cellular stress to take preventive measures of cumulative damage^196,199–206^. Interestingly, Pariset et al. highlighted that real-time monitoring of astronauts’ health is important to prevent or mitigate the health problems related to space radiation and microgravity. Authors proposed that the genotoxic stress in astronauts could be monitored by the quantification of the amount of DNA double-strand breaks (DSBs) in immune cells from the blood of a finger prick. This study observed that individuals with low baseline DSB have fewer clinical complications and enhanced DNA damage repair responses. DSB estimation may be used to predict resilience to spacer radiation^207^. Additionally, plasma vitamin C and erythrocyte GSH could be measured to identify the lack or excess of particular antioxidants to provide supplementation or limit their use^208^. Implementing the blood, serum, and plasma analysis of ROS, oxidative damage, ccf-mtDNA, and DSBs could result in a viable routine analysis inflight thanks to the new and available technology.

Taking a personalized antioxidant cocktail by crew members before, during, and after spaceflight missions surges as a possible option to prevent long-term cumulative effects of ROS damage in cells. Interestingly, it has been observed that redox intraindividual variability exists, and the levels of endogenous antioxidants such as SOD, GSH, GPx, exogenous antioxidants such as vitamin C, and oxidative stress markers are different among individuals and variability could be accentuated during spaceflights as humans respond differently to stress^109,131,208^. An individual stratification of the levels of production of antioxidants based on GWAS, nutritional genomics, and nutrigenetics could help to identify responsive phenotypes that predict nutritional needs and compensate for the activation of pathways that respond to oxidative stress^19,56,208,209^. This information may be used to determine and provide personalized antioxidant cocktail supplementation and define dosage, upper limits, and what type of antioxidant should be supplemented to astronauts.

The maintenance of adequate antioxidant levels, endogenous and exogenous, play an important role in supporting the activity of the immune system, especially when defending our body from viral infections mediating their reactivation. HSV reactivation in astronauts could be an early sign of the damaging effects of increased ROS levels and environmental stress on the immune system. The reactivation of HSV has been observed in missions from 10 to >180 days in shuttles and ISS, respectively. Changes in the levels of the stress hormones (cortisol, dehydroepiandrosterone, epinephrine, and norepinephrine), and a decreased cell-mediated immunity may have contributed to the reactivation of HSV in the crew^210,211^. It has been proposed that inadequate intake of vitamins such as A, C, B complex, and E, among others, decreases our resistance to viral infections and increases disease burden^126,212^. It is reasonable to think that a higher intake of antioxidants than those recommended by the RDA may help to support the immune system of populations with high stress or exposure to environmental pollution, and this may include astronauts. However, clinical evidence is needed to show safety and effectiveness of higher antioxidant doses to prevent or treat cellular and systemic stress during biologically stressful conditions on Earth and space^126,127^. Based on the information presented in the previous section, we propose a cocktail of antioxidants and their doses that could be considered for further research and evaluation of its effectiveness on space missions and travel. The purpose of this section is to encourage the scientific and medical community to understand the efficacy and effectiveness of the proposed doses, modify them, add new antioxidants in order to make space travel safer, and allow longer missions without detrimental effects on our health.

Stratification of astronauts based on the levels of endogenous and exogenous antioxidants in their body from low to high (Table 1). Dosage and quantity by day is proposed for each type of astronaut, based on the reviewed literature and the supplementary tables in the previous section. Astronauts and space travelers with low antioxidant protective levels may need higher doses of antioxidants; in contrast, astronauts with high antioxidant levels may take small doses or those recommended in RDA. Continuous monitoring of antioxidant levels may help in the supplementation and preventive use of high antioxidant doses, helping astronauts recover from facing stressful conditions. Recommended doses are proposed for adults between 19 and 50 years. More stratified groups could be made after studying astronauts’ antioxidant profiles, genetic background, and medical status. It has been previously observed that individuals with low levels of circulating vitamin C and GSH respond better to supplementation after stress^19^.

**Table 1:** Proposed antioxidants cocktail^19,127,208,212,215–263^.

Stratification of astronauts and space travelers accordingly to the exogenous and endogenous antioxidant levels (GWAS, Nutrigenomics Vit C, and GSH assessment)	Low antioxidant protective levels	Intermediate levels	High antioxidant levels, male/female
Vitamin/antioxidant doses based on the astronaut	2 doses per day: 1 every 12 h	1 dose per day	1 dose per day
Vitamin A	1000 mg	50 mg/day	900/700 µg
Vitamin C	4–8 g	1–2 g	90/75–200 mg
Vitamin E	800 to 1200 mg	60–800 mg	15 mg
Vitamin D	50 µg	15–20 µg	15–20 µg
Selenium as L-SeMeT	400 µg	200 µg	55 µg
Resveratrol	1000–1500 mg	500 mg	10 mg
Isorhamnetin	100 mg/kg	100 mg/kg	20–50 mg/kg
Luteolin	100 mg/kg	50 mg/kg	50 mg/kg
CoQ10	1200 mg	20/mg/kg	12/mg/kg

**Fig. 4:**
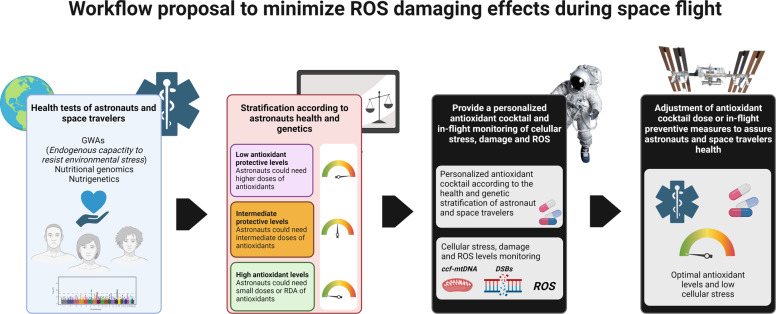
Workflow proposal. In order to minimize the damage of long-term spaceflights and missions to Mars and beyond, it is of great importance to assess the health state and endogenous capacity of astronauts and future space travelers to resist ROS damage. This information will help to stratify the health and endogenous capacity of astronauts to resist environmental and psychological stress of future crew members and provide personalized antioxidant cocktails to prevent ROS and other forms of damage. Monitoring inflight ROS levels, cellular damage, and stress could help to apply preventive or protective strategies specially when astronauts could be exposed to high levels of ionizing radiation such as those experienced during deep-space missions. Continuous monitoring of health during space travel will help to prevent health problems and improve the security of space exploration. Created with BioRender.com.

The proposed workflow includes the assessment of endogenous or exogenous antioxidant levels by GWAS, nutrigenomics, nutrigenetics, and health assessment of risk factors of professional astronauts and space tourists. Stratification of astronauts and dosification of an antioxidant cocktail was according to their group before mission. The length of treatment should be investigated in order to restore the antioxidant levels of astronauts that may have been allocated in the low antioxidant group. It is important to note that, before applying any antioxidant cocktail, the mix of agents and dosage should be properly tested as pro-oxidant results may occur^121^. On-board analysis should be done of ROS, oxidative damage, antioxidant levels, ccf-mtDNA, and DSB. As mentioned previously, we recommend to perform an antioxidant check each 7 days. Knowledge of particular conditions in which ROS production may increase during spaceflights could be important in order to increase antioxidant cocktail dosage that may prevent damage. This series of steps are a recommendation based on the presented information that should be investigated for its safety, efficacy, and plausibility (Fig. 4).

## Conclusion

Spaceflight missions will last longer in the near future as humanity aims to explore Mars and other planets. Astronauts and space explorers will have to face microgravity and high radiation levels for extended periods of time, which could pose major threats to their health^213^. During space missions, an increase in ROS quantities due to microgravity and radiation exposure beyond normal levels can affect the cells, tissues, and organs of astronauts. Excess ROS can accelerate our biological age, by causing musculoskeletal degradation and damages to internal organs and neurophysiology. In order to address this problem, we suggest and highlight in this perspective review the importance of establishing GWAS diagnostics for astronauts in order to better comprehend their genetic susceptibility to ROS damage. Understanding each astronaut’s genetics and capacity to produce endogenous antioxidants could lead to individualized preventive medicine and the personalized administration of antioxidants. This will help prepare astronauts for and treat astronauts after they face the extreme environment of space.

The evidence of antioxidant use on Earth in the prevention of chronic diseases is still a matter of debate; however, as exposure to microgravity and radiation is persistent during space missions, the administration of exogenous antioxidants and the improvement of endogenous production is important to mitigate ROS damage and prevent disease when the crew returns to Earth. The upper limit and the optimal doses of antioxidants for the maintenance of health on Earth and space still need to be determined as variability exists among individuals^208^. There are variations on the requirements and how antioxidants are processed that are influenced by genetic backgrounds and even ethnicity. These variations could be greater during space travel as many physical factors could intervene in the metabolism of drugs or endogenous antioxidant protection^125,214^. Changes of fluid body distribution could alter the absorption of pharmacological compounds and tissue distribution. It has been observed in animal studies that medications are metabolized differently in microgravity^118^. Additionally, physiological changes regarding muscle and bone cellular constitution could alter the pharmacodynamics of therapeutic drugs^118^.

It is of crucial importance to use nutritional genomics and nutrigenetics to predict nutritional needs and aid in the prevention of diseases, such as cardiometabolic, neurodegenerative, and mitochondrial diseases^209^. In order to develop an antioxidant cocktail to prevent ROS damage, it is important to understand how these drugs work and how they could be administered in space, taking into consideration the differences of the genetic background, pharmacodynamics, and pharmacokinetics among individuals and how they could differ on Earth and in space. During spaceflight, astronauts are exposed to high levels of radiation. It is possible to suggest that the dosage of an antioxidant cocktail should reach supranormal levels in order to mitigate excessive cumulative damage. Antioxidants could be exogenously administered not only to restore cell deficits and maintain normal physiology but also to prevent the accumulation of damage due to increased levels of ROS. The scientific knowledge on this topic is continuously evolving, as new exogenous antioxidants are being developed with promising results from in vitro studies, in vivo studies, and in clinical trials. New evidence on the use of antioxidant cocktails with different types and concentrations is urged to be evaluated in order to provide personalization and preventive strategies against the damaging effects of ROS. It will be important to consider these key points to develop better translational strategies and apply the discoveries on Earth to applications in space that will both increase astronaut health and mission length.

### Reporting summary

Further information on research design is available in the Nature Research Reporting Summary linked to this article.

## Supplementary information

Supplementary Information

Reporting Summary

## Data Availability

The corresponding author can provide any data or respond to questions regarding this publication on request.
