# Supplementary material for: Key points for the development of antioxidant cocktails to prevent cellular stress and damage caused by reactive oxygen species (ROS) during manned space missions
Source: NPJ Microgravity. 2021 Sep 23;7:35. doi: 10.1038/s41526-021-00162-8 (PMC8460669; doi:10.1038/s41526-021-00162-8)
Supplement: Supplementary file 1 — Supplementary Information [file 41526_2021_162_MOESM1_ESM.pdf]

Supplementary table 1: Uses of vitamin C in diseases

Disease	Dosage
Skin Bleeds[39]	200mg/day
Scurvy[39]	200mg/day
Upper Respiratory Infections[39]	1000 to 2000mg/day
Chédiak–Higashi Syndrome[39]	1000 to 2000mg/day

Supplementary table 2: Results of the use of vitamin C in randomized clinical trials and other research papers.*

Cardiac and respiratory system		
**Disease**	**Dosage**	**Evidence**
Prevention of cardiovascular disease[131]	No limit was placed on the dose or frequency of vitamin C taken.	There is not enough evidence to suggest that vitamin C supplementation reduces the risk of cardiovascular disease (CVD) in healthy participants and those at increased risk of CVD. Some studies that showed beneficial effects of vitamin C supplementation contained little or very low-quality evidence.
Diabetic retinopathy[142]	The dose used in the study is not specified.	There is not enough evidence to indicate that vitamin C has a significant impact on the progress of diabetic retinopathy.
Lung disease in Cystic Fibrosis (CF)[143]	Not specified due to being provided in a mixed supplement.	Outcome not provided. Additionally, the controls showed improvement in the quality of life compared to children with CF who were administered vitamin supplements.
**Musculo-skeletal system**		
**Disease**	**Dosage**	**Evidence**

Recovery from demanding exercise[144]	200 mg twice a day for 3 days	There is not enough evidence that demonstrates a benefit from the administration of vitamin C after exercise. This may be because this antioxidant cannot be delivered with enough convenience to improve recovery.
Lipid profile and muscle damage in male athletes[145]	200 mg	It has been shown to reduce induced muscle damage and reduce the percentage of body fat.
Antioxidant defense and stress proteins in human lymphocytes and skeletal muscle[146]	500 mg/day for 8 weeks	Vitamin C supplementation has been shown to raise the 70 kilodalton heat shock proteins (Hsp70s or DnaK) that protect the cell from stress so it may be beneficial. However, its long-term effect is not yet known.
**Immunology**		
**Disease**	**Dosage**	**Evidence**
Asthma and exercise-induced broncho-constriction[147]	The dose used in the study is not specified (daily intake).	There is not enough evidence that the use of vitamin C has a significant impact on the management of asthma or exercise-induced broncho-constriction. Some studies suggest a beneficial result in regards to lung function and symptoms, however, they were small studies and they were inconclusive.
Asthma and exercise-induced broncho-constriction[148]	Asthma: 1000 mg/ day for 2 weeks (mild) or 2000 mg/day for 12 weeks (severe)<br><br>EIB: 500 mg/day for 3 weeks	The aim of the study was to determine if there are benefits to using a mix of vitamin C and vitamin E when treating asthma and exercise-induced broncho-constriction. There are no conclusive results about the use of vitamin C in these situations.
Prevention and Treatment of Pneumonia[149]	The dose used in the study is not specified.	The current evidence does not support the use of vitamin C to prevent or treat pneumonia. However, the supplementation of

		vitamin C is still advised in people with vitamin C deficiencies in blood plasma.
Prevention and Treatment of Pneumonia[150]	Prevention: 500 mg/day for 14 weeks; 2 g/day for 8 weeks, 2 g/day for 12 weeks<br><br>Treatment: 125 mg/day until discharge, 200 mg/day for 4 weeks; 200 mg/day until discharge	There are not enough studies to conclude that the use of vitamin C is beneficial for the prevention or for the treatment of pneumonia. More high-quality studies are suggested.
Prevention and Treatment of Common Cold[151]	1 g/day to 2 g/day	High-quality evidence shows that the use of vitamin C fails to prevent or treat the common cold. Thus, the supplementation of vitamin C is not justified in this case.
	**Aging**	
**Disease**	**Dosage**	**Evidence**
Age-related macular degeneration[152]	500 mg/day. Duration 6.3 years	Moderate. Based on a large study of a well-nourished American population, the participants may have experienced a delay in the progression of the disease.
Age-related cataracts[129]	500 mg/day, 3 times a week. Duration 5 years	There is no evidence from randomized clinical trials that supplementation with antioxidant vitamins slows or prevents the progression of age-related cataracts.
	**Neurology**	
**Disease**	**Dosage**	**Evidence**
Charcot-Marie-Tooth disease (CMT)[153]	500 mg/day extended at least 12 successive months	High-quality evidence indicates that vitamin C does not improve the course of CMT1-A in adults in terms of the outcome parameters used.
Prevention and Treatment of	1000 mg/day	The use of vitamin C to prevent and treat tetanus was tested in a single non-

Tetanus[154]		randomized, unblinded, and controlled trial. Thus, there is no reliable evidence to support the use of vitamin C in this case.

*The studies were done under normal conditions on Earth. Space conditions and the constant exposure to stressors could be an important factor to produce different results.

Supplementary table 3: Uses of vitamin E in diseases*

Disease	Dosage
Immunodeficiencies[39]	60 to 800 mg/day
Abetalipoproteinemia[39]	800 to 1200 mg/day
Retrolenticular Fibroplasia[39]	800 to 1200 mg/day
Alzheimer[159]	1340 mg/day

*The studies were done under normal conditions on Earth. Space conditions and the constant exposure to stressors could be an important factor to produce different results.

Supplementary table 4: Results of the use of vitamin E in randomized clinical trials and other research papers.*

Cardiac and Respiratory Systems		
**Disease**	**Dosage**	**Evidence**
Chronic Obstructive Pulmonary Disease (COPD)[160]	402 mg every other day	A women's health study that was randomized, double-blind, placebo-controlled with about 38,600 patients concluded that there was a 10% reduction of chronic lung disease in women
Cardiovascular Disease[161]	Natural: 400-800 mg/day<br><br>Synthetic: 30-600 mg/day	A meta-analysis that encompasses 7 large-scale randomized trials concluded that 6 out of 7 trials showed that Vit. E had no statistically nor clinically significant effect on cardiovascular disease

Musculo-skeletal system		
**Disease**	**Dosage**	**Evidence**
Muscle cramps in patients with cirrhosis[162]	**Drug: Vitamin E**<br>266 mg of vitamin E nightly for 3 months.<br>**Drug: Ropinirole**<br>0.5 mg ropinirole nightly for 3 months.	This was a randomized phase four intervention study involving 60 people. The objective was to compare the efficacy of Ropinirole, a dopamine agonist, with vitamin E in the treatment of muscle cramps in cirrhotic patients. The results in this study were not published so the long-term effects are not known.
Muscle cramps in amyotrophic lateral sclerosis[163]	800 IU alternating with placebo	This was a randomized phase three intervention study involving 32 participants. A placebo was also used in the study and the participants were divided into two groups. Group A received only vitamin E and group B received the placebo. After the seventh week, group A began receiving placebo and B vitamin E. The results showed a reduction in the number of cramps and duration of muscle cramps experienced over a two week period.
Pain, oxidative stress and inflammatory response after resistance exercise (vitamin E and vitamin C supplementation combined with cryotherapy were used)[164,165]	Single doses of vitamin C: 1000 mg and vitamin E: 800 IU	This is a randomized Clinical study evaluating 14 participants that underwent 4 sessions of resistance exercise with distinct forms of recovery. Recovery with vitamin c and vitamin e along with cryotherapy produced decreased pain and inflammatory response during recovery.
**Immunology**		
**Disease**	**Dosage**	**Evidence**
Respiratory infection in elderly[166]	133.4 mg/ day of alpha-tocopherol for 1 year.	A randomized, double blinded control trial with a total of 617 patients ages 65 or older concluded that supplementation

		with vitamin E did not show a significant effect on lower respiratory infections. However, it showed a protective effect on upper respiratory infections but further investigations are needed.
Immune response to tetanus toxoid immunization[167]	400 mg tocotrienol-rich fraction (TRF) daily	A double-blind, placebo-controlled clinical trial concluded that supplementation with TRF increased production of interferon γ and interleukin 4. This produces a beneficial effect by stimulating the immune system in healthy subjects and shows possible clinical benefits by enhancing the immune response to vaccines.
Human Immunodeficiency Virus (HIV) Disease progression and Mortality[168]	Standard dose regimen: 15 mg/day<br><br>High dose regimen: 30 mg/day	A randomized, double-blind, controlled trial of high dose vs standard dose multivitamin supplementation was performed in Tanzania during 24 months using patients with HIV who received highly active antiretroviral therapy (HAART). The multivitamin supplementation contained vitamin E, vitamin C, folic acid, vitamin B12, niacin, vitamin B6, riboflavin and thiamine. This study concluded that a high dose multivitamin supplementation did not show a change in progression and risk of mortality of (acquired immuno-deficiency syndrome) AIDS compared to the standard dose. However, the high dose regimen caused a significant increase of alanine transaminase levels.
**Aging/Neurology**		
**Disease**	**Dosage**	**Evidence**
Skin aging[169]	6.67 mg/day for 12 weeks of D-alpha-Tocopherol.	This study was a clinical interventional, randomized, double-blind and placebo-controlled trial with 100 patients. It

		aimed to evaluate the oral supplement containing Astaxanthin, Lycopene, and D-Alpha-Tocopherol. A scale classification from 0 (no wrinkles) to 4 (severe wrinkles) was used. At week 12, the subjects presented a significant increase in skin hydration (measured by corneometer) and decrease in pigmentation registered by mexameter.
Age-related macular degeneration[152]	50 mg/day<br>266.8 mg/alternate days<br>400.2 mg/alternate days<br>333.5 mg/day	This review analyzed 5 randomized clinical trials conducted in Australia, Finland, and the United States which investigated whether diets rich in antioxidant vitamins (including vitamin E) or minerals can delay macular degeneration caused by age, by about 4 - 10 years. Evidence showed that vitamin E supplements do not prevent development of macular degeneration and may even increase the risk of delayed production. For this reason, the authors consider that more evidence is required to be clear about the benefits of vitamin E before recommending its use.
Alzheimer’s dementia and mild cognitive impairment (MCI)[170]	Capsules 1,334 mg/day in 2 divided doses.	This study evaluates the efficacy of vitamin E in the treatment of these diseases. Double-blind randomized trials were analyzed and then compared with a placebo for approximately 48 months. As a result, the authors concluded that no evidence was found that alpha-tocopherol administered to people with MCI prevents progression of dementia, or that it improves cognitive function. However, 1 study found that it can delay Alzheimer's functional decline. It was also shown that high doses (2010mg/day) are considered toxic and cause fatigue, GI

		cramps, and diarrhea. The average dose was 266.8 mg/day, 1334mg/day was used as the maximum dose.
Epilepsy[171]	294 mg/day for 3 months[172].	This review assessed whether vitamins (including Vitamin E) help control seizures, reduce adverse effects of antiepileptic drugs, and / or improve quality of life. In a study with 24 participants a decrease in seizure frequency was found. However, this study contained a limited number of individuals, therefore more trials are required to further support its usefulness.

*The studies were done under normal conditions on Earth. Space conditions and the constant exposure to stressors could be an important factor to produce different results.

Supplementary table 5: Results of the use of β-carotene in randomized clinical trials and other research papers.*

**Disease**	**Doses**	**Evidence**
Cystic Fibrosis[181]	50 mg/day.	The supplementation of 1000 mg/kg (in patients up to 50kg) and 50 mg/kg (in patients with more than 50 kg) of beta-carotene for 12 weeks, increased the total antioxidative capacity in plasma of the Cystic Fibrosis patients. Thus normalizing the levels of beta carotene and malondialdehyde.
Cystic Fibrosis[182]	18,167 IU/day for 12 weeks (92%β-carotene and 8% palmitate).<br><br>*One of the components of a multivitamin.	The subjects enrolled in this trial showed modest improvements in weight and pulmonary function and increased β-carotene levels were weakly

		associated with better growth parameters. However, it lacks effect on oxidative stress.
Insulin Resistance and Adiposity[183]	3.75 mg/per day during breakfast and dinner.<br><br>*One of the components of a multivitamin.	The supplementation of this multivitamin over a 6 month period was associated with an increase in serum β-carotene concentrations and a reduction in adiposity in conjunction with an improvement in insulin resistance in overweight boys.

*The studies were done under normal conditions on Earth. Space conditions and the constant exposure to stressors could be an important factor to produce different results.

Supplementary table 6: Results of the use of L-selenomethionine in randomized clinical trials and other research papers.*

Disease	Doses	Evidence
Precancerous/ Non Malignant condition Prostate Cancer[191].	200 ug per day for 3 years.	The reduction of the risk in Developing prostate cancer between the patients who were given placebo and L-selenomethionine did not show a significant difference.
Locally-advanced squamous cell carcinoma of the head and neck[192].	Drug: 3600 μg/m2 L-selenomethionine twice daily for 7 days before chemoradiation, once daily during chemoradiation, and for three weeks after chemoradiation [192].<br>Drug: 100mg/m2 cisplatin every three weeks during chemoradiation[192] .	Despite the fact that the addition of L-selenomethionine was well tolerated by the patients, it did not show a decrease in the incidence of mucositis or an improvement in quality of life [192].
Autoimmune thyroiditis[193 194,195].	Organic selenium: 200 μg per day in the form of L-	There was not a significant difference in the applied

	selenomethionine, for 3 or 6 months[193,194]. The clinical trial involved children and adolescents aged 4 to 18 years old [193].	treatment.
Peripartum cardiomyopathy[195].	200 µg/day for 3 months. The trial involved patients who had peripartum cardiomyopathy and selenium deficiency [195].	Selenium supplementation did not reduce the risk of unrecovered left ventricular systolic function or death, but did significantly reduce heart failure symptoms.
Selenium and Vitamin E to treat age-related cataract[196].	200 µg per day of L-selenomethionine and vitamin E, 400 IU per day.	No significant effect on age related cataract prevention.
Vitamin C, vitamin E, zinc gluconate, and selenomethionine supplementation on muscle function and oxidative stress biomarkers in patients with facioscapulohumeral dystrophy[197]	500 mg vitamin C, 400mg vitamin E, 25mg zinc gluconate and 200g selenomethionine per day	In conclusion, vitamin E, vitamin C, zinc, and selenium supplementation had no significant effect on the two minute walking test, but improved maximal voluntary contraction and indurance limit time of both quadriceps by enhancing the antioxidant defences.

*The studies were done under normal conditions on Earth. Space conditions and the constant exposure to stressors could be an important factor to produce different results.

Supplementary table 7: Results of the use of resveratrol in randomized clinical trials and other research papers.*

**Systemic**		
**Disease:**	**Doses:**	**Evidence:**
Inflammation[202]	160 mg/kg	A randomized trial with mice given resveratrol chow, showed that RES reduced the expression of proinflammatory cytokines IL-1, IL-66, and TNF-alpha.
Sepsis-induced DNA damage in the lymphocytes of rats [204]	100 mg/kg	In a study with 32 albino rats, RES increased SOD,

		and GPx activity in the liver of these rats.
**Neurology**		
**Disease:**	**Doses:**	**Evidence:**
Alzheimer's disease [205]	500 mg per day (with escalations of 500 mg increments every 13 weeks until 1000 mg twice daily	The levels of matrix metalloproteinase 9 were significantly reduced after resveratrol treatment. As metalloproteinase 9 regulates the permeability of the blood brain barrier, resveratrol treatment probably reduced central nervous system permeability. Moreover it may induce adaptive immune responses that promote brain resilience to amyloid deposition. More studies are needed to confirm these results.
**Endocrine**		
**Disease:**	**Doses:**	**Evidence:**
Type 2 Diabetes Mellitus (T2DM)[206]	10 mg, 150 mg or 1000 mg for<br>4 to 5 weeks.	There is insufficient evidence for resveratrol supplementation to be used in treatment or evidence to support an effect, either beneficial or adverse, in adults with T2DM comparing the effect with placebo, no treatment, other antidiabetic medications, diet or exercise.<br>There was one clinical trial measuring the effects of resveratrol on endothelial function in T2DM, with no results posted,

		nevertheless, the previous systematic review assessed that clinical trial.

*The studies were done under normal conditions on Earth. Space conditions and the constant exposure to stressors could be an important factor to produce different results.

Supplementary table 8: Results of the use of isorhamnetin in randomized clinical trials and other research papers.*

**Cardiovascular**		
**Disease:**	**Doses:**	**Evidence:**
Anti-atherosclerosis[217]	20 mg /kg	Experiments were conducted in mice fed with a high-fat diet. Isorhamnetin reduced ROS levels and macrophage apoptosis, and inhibited the formation of atherosclerotic plaque in mice by affecting the PI3K/AKT and NF-κB signaling pathways. Additionally, experiments showed that the use of 100 mg/kg/day of isorhamnetin inhibited the PI3K/AKT signaling pathway and reduced myocardial hypertrophy and fibrosis caused by pressure load. Therefore, isorhamnetin also has the potential to prevent myocardial hypertrophy. However, specific mechanisms need further validation.
Myocardial Hypertrophy[217]	100 mg/ kg / day	
**Neurology**		
**Disease:**	**Doses:**	**Evidence:**
Neurodegenerative	50 mg / kg	Experiments were

Diseases[217]		conducted in albino mice with an intra-abdominal injection of scopolamine (3 mg / kg). It was shown that isorhamnetin can attenuate scopolamine-induced activity of cholinesterase and brain-derived neurotrophic factor in the prefrontal cortex and hippocampus enhances cholinergic signaling and synaptic plasticity. Therefore, isorhamnetin can be developed as an anti-acetylcholinesterase reagent to prevent neurodegenerative diseases.
	**Hepatology**	
**Disease:**	**Doses:**	**Evidence:**
Hepatic fibrosis[217]	30 mg / kg	Experiments were conducted on mice from the Institute of Cancer Research that were stimulated by carbon tetrachloride. Isorhamnetin suppresses the activation of hepatic stellate cells (HSC) through the activation of NRF2-ARE signaling. The main function of HSC is the production of extracellular matrix and collagen, therefore, inhibiting these cells prevents liver fibrosis.
	**Bone**	
**Disease:**	**Doses:**	**Evidence:**
Osteoporosis[217]	30 mg/ kg / day	Experiments were conducted in ovariectomized Sprague-

		Dawley rats. Isorhamnetin inhibits osteoclastogenesis and bone resorption in rat femoral shaft and tendon tissue and mouse bone marrow cells to prevent osteoporosis. However the mechanism of action still remains unclear, and thus further investigation is required.

*The studies were done under normal conditions on Earth. Space conditions and the constant exposure to stressors could be an important factor to produce different results.

Supplementary table 9: Results of the use of luteolin in randomized clinical trials and other research papers.*

**Neurology**		
**Disease:**	**Doses:**	**Evidence:**
Alzheimer's Disease[218]	5, 10, 15, and 20μM doses of Luteolin (mixed in diet) for 30 days.<br><br>*Dose used as part of the diet.<br>Diet not specified.	Experiments were performed in transgenic Drosophila expressing Aβ42 (beta amyloid). Accumulation of Aβ-42 peptides induces oxidative stress and induces signaling pathways leading to neuronal cell death. Luteolin was shown to reduce oxidative stress and accumulation of Aβ42 peptides in flies with Alzheimer's. The study suggests that luteolin could be used as a powerful pharmaceutical agent in the treatment of Alzheimer's disease.
**Immunology**		
**Disease:**	**Doses:**	**Evidence:**

Psoriasis[219]	50 mg / kg*<br><br>*As a component of multi-therapy<br>Single dose injected intraperitoneally.	Experiments were performed in mice as models of psoriasis. Efficacy in reducing injuries was demonstrated. This indicates its potential for clinical use in psoriasis treatments. However, the efficacy of luteolin in decreasing the action of HSP90 (key regulator of psoriasis inflammation) may also be associated with the internal environment and other complex factors, requiring further investigation.
**Hepatology**		
**Disease:**	**Doses:**	**Evidence:**
Non-alcoholic fatty liver disease[220]	20 or 100 mg/kg/ day for 8 weeks.	Experiments were performed in mice as models of NAFLD. Efficacy in improving hepatic steatosis associated with obesity was demonstrated. Luteolin can suppress the hepatic conversion of excess carbohydrates to triglycerides by directly blocking the LXR-SREBP-1c pathway, which integrates hepatic glucose metabolism and fatty acid synthesis. Therefore, the study suggests that luteolin may have future utility in the clinical setting as a treatment for NAFLD.

*The studies were done under normal conditions on Earth. Space conditions and the constant exposure to stressors could be an important factor to produce different results.

Supplementary table 10: Results of the use of CoQ10 in randomized clinical trials and other research papers

Cardiovascular		
**Disease:**	**Doses:**	**Evidence:**
Heart Failure[240]	60-300mg	A meta-analysis of 13 studies concluded that there was a significant net improvement in the fraction ejection ; CoQ10 may be of benefit to patients with Congestive Heart Failure.
**Hepatic**		
**Disease:**	**Doses:**	**Evidence:**
Metabolic Syndrome and Non-Alcoholic Fatty Acid Liver Disease[241]	100 mg/day	A randomized, double blind, placebo-controlled 4-week study with CoQ10 showed reductions of liver enzymes and abdominal circumference in patients that suffered from NAFLD.
**Endocrine**		
**Disease:**	**Doses:**	**Evidence:**
Type 2 Diabetes Mellitus[242]	200 mg	A randomized, double blind, placebo-controlled 12-week study of patients with T2DM, showed a decrease in both fasting glycemia and glycated hemoglobin.

*The studies were done under normal conditions on Earth. Space conditions and the constant exposure to stressors could be an important factor to produce different results.
